# Targeting Cell Signaling Pathways in Lung Cancer by Bioactive Phytocompounds

**DOI:** 10.3390/cancers15153980

**Published:** 2023-08-05

**Authors:** Neeraj Choudhary, Sweta Bawari, Jack T. Burcher, Dona Sinha, Devesh Tewari, Anupam Bishayee

**Affiliations:** 1 Department of Pharmacognosy, GNA School of Pharmacy, GNA University, Phagwara 144 401, India; 2 Amity Institute of Pharmacy, Amity University, Noida 201 301, India; 3 College of Osteopathic Medicine, Lake Erie College of Osteopathic Medicine, Bradenton, FL 34211, USA; 4 Department of Receptor Biology and Tumor Metastasis, Chittaranjan National Cancer Institute, Kolkata 700 026, India; 5 Department of Pharmacognosy and Phytochemistry, School of Pharmaceutical Sciences, Delhi Pharmaceutical Sciences and Research University, New Delhi 110 017, India

**Keywords:** lung cancer, phytochemicals, signaling pathways, preclinical, clinical studies

## Abstract

**Simple Summary:**

Lung cancer is the leading cause of mortality in cancer patients, causing an estimated 1.8 million deaths in the year 2020. However, the available therapeutic options exert numerous adverse effects and adequate therapeutic activity is still to be achieved. Therefore, there is a need for the development of safe and effective treatment for lung cancer. Phytochemicals are well documented for their anticancer potential against lung cancer and have a strong rationale for further investigation as a potential chemotherapeutic agent. Notably, phytochemicals act by modulating several signaling pathways, promoting apoptosis, oxidative stress, and disruption of the mitochondrial membrane, inhibiting angiogenesis, and regulating transcription factors. Therefore, an exhaustive and detailed review was carried out to establish the potential role of phytochemicals by conducting a critical analysis of in vitro, in vivo, and clinical evidence in mitigating lung cancer, with emphasis on their impact on signaling pathways.

**Abstract:**

Lung cancer is a heterogeneous group of malignancies with high incidence worldwide. It is the most frequently occurring cancer in men and the second most common in women. Due to its frequent diagnosis and variable response to treatment, lung cancer was reported as the top cause of cancer-related deaths worldwide in 2020. Many aberrant signaling cascades are implicated in the pathogenesis of lung cancer, including those involved in apoptosis (B cell lymphoma protein, Bcl-2-associated X protein, first apoptosis signal ligand), growth inhibition (tumor suppressor protein or gene and serine/threonine kinase 11), and growth promotion (epidermal growth factor receptor/proto-oncogenes/phosphatidylinositol-3 kinase). Accordingly, these pathways and their signaling molecules have become promising targets for chemopreventive and chemotherapeutic agents. Recent research provides compelling evidence for the use of plant-based compounds, known collectively as phytochemicals, as anticancer agents. This review discusses major contributing signaling pathways involved in the pathophysiology of lung cancer, as well as currently available treatments and prospective drug candidates. The anticancer potential of naturally occurring bioactive compounds in the context of lung cancer is also discussed, with critical analysis of their mechanistic actions presented by preclinical and clinical studies.

## 1. Introduction

Lung cancer represents one of the most frequently diagnosed malignancies globally, falling behind only prostate cancer in males and breast cancer in females [1,2]. As most cases are discovered as locally advanced or metastatic disease, lung cancer has a notoriously poor five-year survival rate (18.6%) compared with other cancers, such as colorectal (64.5%), breast (89.6%), and prostate (98.2%) [2,3,4]. Depending on disease progression and patient goals, several treatment options may be used against lung cancer, including chemotherapy, radiotherapy, immunotherapy, and surgical intervention [5]. Historically, broad-spectrum chemotherapy has been the mainstay of treatment, but targeted therapies have begun to emerge as highly efficacious options, thereby shifting focus towards personalized medicine. This advancement is made possible by extensive and ongoing research on the cellular signal transduction pathways disrupted in lung malignancies [6].

Despite new therapies and ongoing developments, cancer of the lung was deemed the number one cause of cancer-related deaths worldwide in 2020, claiming an estimated 1.8 million lives [7]. Unfortunately, current therapeutic options are not providing adequate response and are frequently accompanied by significant adverse effects [2]. Approved agents routinely used in medical oncology for the treatment of lung cancer are notorious for their toxic effects. Many first-line drugs are documented to have toxicity such as celecoxib [8], carboplatin, or cisplatin in combination with oxaliplatin [9] and docetaxel [10]. The need for novel, safe, and effective treatments for lung cancer is further underscored in the setting of recurrent and drug-resistant cancers [3,4]. Ultimately, the high mortality and frequent adverse effects of classic treatments serve as an impetus to explore medicinal plants for their pharmacological beneficence.

Plant-based metabolites have been shown to possess anticancer activity in the context of lung malignancies [11]. Furthermore, various challenges associated with the effective and safe use of plant metabolites have been overcome due to the new approaches used in the pharmaceutical industry [12,13,14]. Specialized plant-derived metabolites are strong contenders as anticancer drugs due to their reduced toxicity and high efficacy against lung cancer [15]. Plant metabolites exert their anticancer activity via different mechanisms in lung cancer but act primarily by inhibiting cellular metabolism, thereby preventing tumor cell proliferation [16,17]. Previous reviews have attempted to capture the broad scope of phytochemicals in the context of lung cancer. One review highlights the structure–activity relationship of various bioactive compounds in regard to non-small cell lung cancer (NSCLC) only and failed to provide significant insight on mechanisms of action [18]. In another review, the role of natural products was discussed in the context of lung cancer, but this study’s discussion of phytochemicals was limited to their targeting of the tumor microenvironment [17]. Another article discusses the anticancer effects of various phytochemicals in lung cancer stem cells but was limited in scope by its selection of only nine phytochemicals and emphasis on stem cells [19]. In one more publication, the roles of a few phytochemicals were discussed against lung cancer biomarkers [20]. Still, limitations in scope are seen in other publications by narrowing inclusion criteria to only one group of phytochemicals, e.g., phenolics [21]. Numerous phytochemicals were recognized, and their mechanisms of action were explained. However, the review was not utterly comprehensive, and since then, several in vitro, in vivo, and clinical studies were conducted in the past few years, which have identified numerous additional phytochemicals that displayed anticancer effects in lung cancer [16]. An exhaustive and detailed review of the role of phytochemicals against lung cancer, including a focused discussion of their mechanistic action as presented in preclinical and clinical studies, is still lacking. Therefore, this review is an attempt to provide an up-to-date discussion of all phytochemicals relevant to the treatment or prevention of lung cancer, with emphasis on their impact on signaling pathways.

## 2. Pathophysiology of Lung Cancer

**Figure 1:**
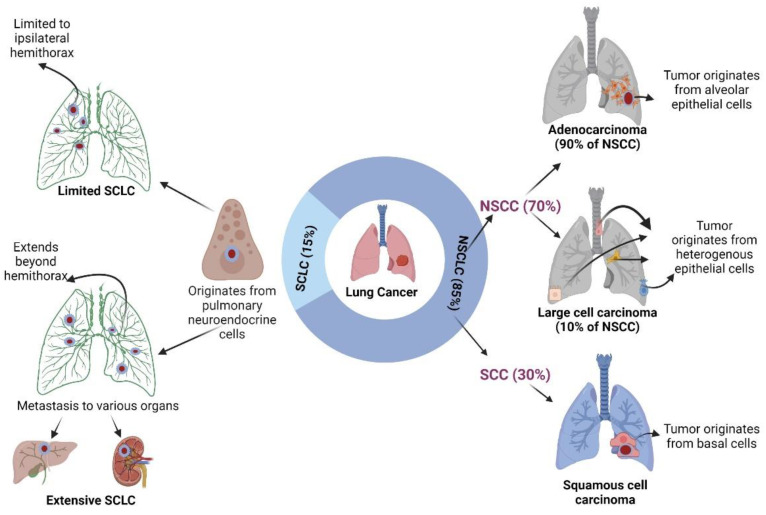
Various types of lung cancer. Lung cancer is categorized into small cell lung cancer (SCLC) which accounts for 15% of all lung cancers and non-small cell lung cancer (NSCLC) which accounts for the remaining 85%. SCLC originates from pulmonary neuroendocrine cells and is further categorized into limited SCLC, characterized by involvement of the ipsilateral hemithorax, and extensive SCLC, which extends to the contralateral hemithorax or beyond. NSCLC is subdivided into squamous cell carcinoma (SCC), which comprises 30% of all NSCLC cases and originates from lung basal cells, and non-squamous cell carcinoma (NSCC) that encompasses 70% of all NSCLC incidences. Ninety percent of cases of NSCC are those of adenocarcinoma that originates from alveolar epithelial cells; only 10% of cases of NSCC are those of large cell carcinoma which exhibits tremendous heterogenicity in its origin.

The World Health Organization (WHO) broadly divides lung cancers into two major categories: small cell lung cancer (SCLC) and NSCLC [22]. Of the two, NSCLC is more prevalent and constitutes approximately 80% of lung cancers. SCLC, however, is more aggressive, develops rapidly, and is more prone to metastasis compared to NSCLC [23,24]. Whereas SCLC is more central in location with its common site of origin in the bronchial epithelium [22], NSCLC is more peripheral and essentially originates in the epithelium of either bronchioles or alveoli [25]. Moreover, SCLC originates from neuroendocrine cells, whereas NSCLC originates from various types of epithelial cells [26]. NSCLC is further divided into three histological groups, including centrally located squamous cell carcinoma, distally located adenocarcinoma, and large cell lung cancer, which is variable in its location [3,26,27]. Similarly, SCLC is categorized into limited and extensive types based on if the confined borders of SCLC are limited to the ipsilateral hemithorax and the associated lymph nodes or if there is any spread of the malignancy to areas beyond the thorax [24,28] (Figure 1). Regardless of the varying subtypes, all lung cancers follow a similar course of events stemming from genetic mutations which usually occur following exposure to carcinogens, eventually followed by clonal expansion of the implicated cells [29].

**Figure 2:**
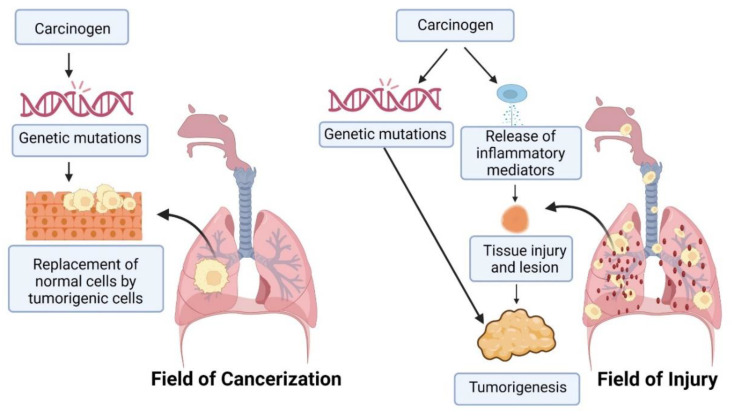
Field of cancerization and field of injury theories of lung cancer development. Field of cancerization is based upon the replacement of normal pulmonary cells by tumorigenic cells upon exposure to carcinogens. These cells go on to produce genetic aberrations, ultimately leading to carcinogenesis in the exposed areas of the lungs. Field of injury-mediated tumorigenesis is an outcome of carcinogen-induced genetic mutations and tissue injury as a result of extensive host response. These carcinogen-induced tumorigenic lesions are not restricted to a particular region or field (as in field of cancerization) but are widespread in the entire respiratory tract and lungs.

Two major hypotheses have been put forth to best describe the pathogenesis of lung cancer, namely “the field of cancerization” and “the field of injury” theories (Figure 2) [27]. Field of cancerization is based upon observations made by Slaughter in the year 1944 [30], later expanded upon by Auerbach et al. [31] who reported serious histological changes in the bronchial epithelium as a result of cigarette smoking in 1957. According to this theory, carcinogens induce extensive genetic aberrations that can be seen in the regions with a neoplasm and immediately adjoining respiratory epithelium, more like involving a patch or field of histological changes that develop into cancer [29]. Although there may be no apparent morphological changes, the normal cells in a field of cancerization are replaced by tumorigenic cells owing to carcinogen-induced genetic mutations long before an actual cancerous lesion develops [32]. Therefore, even after the tumor develops and is surgically removed, the field of cancerization remains, resulting in secondary tumorigenesis [33]. The field of injury describes extensive histological changes that occur throughout the carcinogen-exposed areas of the respiratory tract, including the epithelial tissues in the airway, as well as the lungs, suggesting host response upon carcinogen encounter [34]. This host response is responsible for inflammation and genetic aberrations which may result in the genesis of a neoplasm [30].

NSCLC is majorly an outcome of Kirsten rat sarcoma viral oncogene (KRAS) mutations and epidermal growth factor receptor (EGFR) mutations. Other genes which may harbor mutations and contribute to NSCLC in some capacity include anaplastic lymphoma kinase (ALK), mesenchymal epithelial transition factor (MET), V-Raf murine sarcoma viral oncogene homolog B (BRAF), mitogen-activated protein kinase (MAPK) or extracellular signal-related kinase (ERK) kinase (MEK), and rearranged during transfection (RET) mutations. In contrast, gene alterations most typically seen in the setting of SCLC include mutations of phosphatidylinositol-4,5-bisphosphate 3-kinase catalytic subunit alpha (PIK3CA), fibroblast growth factor receptor 1 (FGFR1), and phosphatase and TENsin homolog gene (PTEN) [35], which are addressed in subsequent sections. Inactivating mutations in the tumor suppressor TP53 and retinoblastoma (RB) 1 genes are witnessed ubiquitously in approximately 90% of the cases of SCLC [36,37]. Similarly, inactivation of RB tumor suppressor gene is also a very common finding in SCLC [38]. These genetic mutations in NSCLC and SCLC are considered to be the major oncogenic drivers due to their ability to affect various upstream and downstream signaling molecules of numerous pathways, including the phosphatidylinositol-3-kinase (PI3K)/protein kinase B (Akt)/mammalian target of rapamycin (mTOR) pathway (PI3K/Akt/mTOR pathway); rat sarcoma virus gene (RAS)/rapidly accelerated fibrosarcoma (RAF)/MAPK or ERK kinase (MEK)/ERK pathway (RAF/MEK/ERK pathway); and Janus kinase (JAK)/signal transducer and activator of transcription (STAT) transduction pathway (JAK/STAT pathway) [35].

## 3. Cell Signaling Pathways in Lung Cancer

Lung cancer is a product of aberrations in normal cell function, including oxidative stress, genetics, and multiple signaling pathways [39,40]. Of the signaling pathways implicated in lung cancer, receptor tyrosine kinases (RTKs) are most frequently involved in carcinogenesis. These transmembrane receptors are further involved in triggering a multitude of signaling cascades that ultimately result in activation of prosurvival oncogenes, such as X-linked inhibitor of apoptosis protein (XIAP), myeloid cell leukemia sequence 1 (Mcl-1), survivin, and B cell lymphoma protein-2 (Bcl-2). Additionally, RTKs promote the inactivation of the proapoptotic genes, such as Forkhead box O (FOXO), further promoting cell proliferation, survival, and cell cycle progression [41,42]. The major signaling pathways implicated in the pathogenesis of lung cancer include RAF/MEK/ERK, PI3K/Akt/mTOR, and JAK/STAT signaling. The detailed role of these pathways is presented in the present section.

### 3.1. RAS/RAF/MEK/ERK Pathway

The RAF/MEK/ERK signal transduction pathway is a major player involved in cell proliferation, apoptosis, and senescence [43,44] and has been shown to be an active participant in both NSCLC [45] and SCLC [46]. RAS encodes a G protein with guanosine triphosphatase activity [47] and acts as a crucial scaffold between the cell surface receptors and various downstream signaling pathways responsible for cell survival and cell proliferation. These cell surface receptors include EGFR and fibroblast growth factor receptor (FGFR). Downstream signaling cascades include the PI3K/Akt/mTOR pathway; RAS/RAF/MEK/ERK pathway [48]; and Ras-like (Ral) guanine nucleotide exchange factors (GEFs)/Ral [49]. Upon binding of growth factors to their respective receptors, RAS is activated with the aid of the growth factor receptor-bound protein 2 (Grb2)/son of sevenless (SOS) coupling complex [50]. RAS then undergoes a conformational change, binds GTP, and further recruits RAF (A-RAF, B-RAF, or RAF-1). Upon binding to the cell membrane, RAF undergoes dimerization, allowing removal of the inhibitory actions of RAF kinase. RAF further associates with proteins such as heat shock protein 90 (HSP90) which aids in stabilizing the RAF dimer [49]. RAF dimer then triggers phosphorylation–activation of downstream MEK (MEK1 or MEK2), which activates ERK (ERK1 or ERK2). ERK undergoes dimerization and translocates to the nucleus to regulate the transcription of genes like c-Fos, c-Jun, c-Myc, CREB, MSK, and ELK-1. These genes are crucial regulators of cell cycle progression and proliferation.

Mutations in KRAS, Harvey rat sarcoma virus (HRAS), and neuroblastoma rat sarcoma viral oncogene homolog (NRAS) oncoproteins of the RAS superfamily have been reported in various cases of NSCLC. Amongst these genes, KRAS is the most frequently mutated, primarily in adenocarcinomas and to a lesser extent in squamous cell carcinoma [48]. Contrarily, KRAS mutations are a rare finding in SCLC [38]. Missense mutation resulting in replacement of glycine with cysteine at codon 12 (KRAS G12C mutation) is the most commonly recorded mutation of KRAS in NSCLC. KRAS mutations are strongly associated with smoking [47]. Unlike KRAS mutations, mutations in BRAF are less frequent and usually found in NSCLC patients who are non-smokers [51].

### 3.2. PI3K/Akt/mTOR Pathway

The PI3K/Akt/mTOR pathway is chiefly responsible for the development and exacerbation of lung cancers. PI3K/Akt/mTOR signal transduction is initiated upon binding of growth factors to the cell surface receptor tyrosine kinases (RTKs). These RTKs include vascular endothelial growth factor receptor (VEGFR), human epidermal growth factor receptor-2 (HER2), insulin-like growth factor receptor (IGFR), epidermal growth factor receptor (EGFR), and platelet-derived growth factor receptor (PDGFR). This ligand–receptor binding triggers localization of PI3K to the plasma membrane via the p85 (regulatory) subunit of PI3K, which binds to the RTK. This is followed by phosphorylation and dimerization of PI3K. Upon binding of the regulatory p85 subunit, the p110 (catalytic) subunit of PI3K is exposed. This p110 subunit then catalyzes the formation of phosphatidylinositol 3,4,5-trisphosphate (PIP3) upon phosphorylation of phosphatidylinositol 4,5-bisphosphate (PIP2). PIP3 serves as a secondary messenger and commences the recruitment of Akt to plasma membrane and its phosphorylation and activation by 3-phosphoinositide-dependent kinase 1 (PDK1) and subsequently by mTOR complex-2. This phosphorylation and activation of Akt results in the dissociation of Akt from the plasma membrane into the cytoplasm where it serves as a crucial mediator of cell proliferation, growth, and survival. Akt produces these effects by phosphorylating and activating downstream effector proteins like mTOR complex-1, 4E-binding protein 1 (4EBP1), and p70S6 kinase 1 (p70S6K). Akt also upregulates prosurvival proteins like X-linked inhibitor of apoptosis protein (XIAP) and Bcl-2 and downregulates proapoptotic proteins, fostering cancer progression and exacerbation. Normally, this is kept in check by phosphatase and tensin homolog (PTEN) tumor suppressor protein in healthy cells, which inhibits the activation of Akt by negatively regulating PIP3, thus halting the PI3K/Akt/mTOR signaling cascade [41,52,53]. But, the PI3K/Akt/mTOR signaling pathway has been reported to be majorly dysregulated in NSCLC [54,55]. This has been witnessed to be the outcome of inactivating mutations of PTEN [56,57]. Also, overexpression of PIK3CA has been reported to be a major driving event in NSCLC development and progression [58,59,60,61]. Over activity of the p110α catalytic subunit of PIP3 as a result of activating mutations majorly in the helical and kinase domain of the PIK3CA gene was found to be a main culprit in lung cancer progression [62].

### 3.3. JAK-STAT Pathway

Janus kinase of the JAK-STAT pathway is constitutively present intracellularly in association with the transmembrane receptors such as interleukin-6 receptor, granulocyte colony-stimulating factor receptor, and erythropoietin receptor [63]. JAK proteins (JAK1-3, tyrosine kinase-2) possess four functional domains, including the FERM domain which serves as a binding site to bind to the receptors; the SH2 unit to bind to phosphorylated tyrosine residues; a phosphorylating JH1 domain; and a regulatory JH2 unit [63,64]. STAT proteins (STAT1-4, STAT5A, STAT5B, and STAT6) possess an N-terminal domain that promotes dimerization for binding to transcription factors; a regulatory coiled-coil domain; DNA-binding domain; an SH2 domain to bind to phosphorylated tyrosine residues; a linker domain that links the DNA-binding domain to the SH2 domain; and a transcription–activation domain [64]. Upon binding of a ligand to the extracellular domain of the JAK-associated receptor, receptor dimerization and conformational changes lead to phosphorylation and activation of JAK. Activated JAK then phosphorylates the tyrosine residues in the intracellular domains of the bound receptor that recruits STAT and induces its phosphorylative activation. STAT, upon activation, dissociates and dimerizes through its SH2 domain and translocate to the nucleus where it regulates the transcription of numerous target genes [63,64] involved in cell proliferation, differentiation, inflammation, and apoptosis [65,66].

The JAK-STAT pathway is kept under check by regulatory proteins. Protein inhibitor of activated STAT (PIAS) blocks STAT-mediated transcription of target genes by inhibiting STAT–DNA interaction. Protein tyrosine phosphatase (PTP) promotes dephosphorylation inactivation of the tyrosine residues of the receptor, JAK, and STAT, whereas suppressor of cytokine signaling (SOCS) is involved in blocking binding sites for STAT at the receptor, in addition to promoting proteasomal degradation of STAT via ubiquitination [63,64,65,67].

### 3.4. NRF2-KEAP1-ARE Pathway

The nuclear factor erythroid 2-related factor 2 (NRF2)–Kelch-like ECH-associated protein 1 (KEAP1)–antioxidant responsive elements (AREs) pathway is basically a protective measure of the cells against reactive oxygen species (ROS) and electrophilic stress signals [68]. NRF2 is a redox regulator from the basic leucine zipper protein family, whereas KEAP1 is an adaptor protein. Under normal conditions, NRF2 undergoes ubiquitination through interaction with Cullin-3 carrying a ubiquitin E3 ligase complex mediated through a KEAP1 scaffold. This marks NRF2 for proteasomic degradation. Under conditions of oxidative and electrophilic stress, KEAP1 sequestration of NRF2 is halted, and released NRF2 then translocates to the nucleus [68,69]. In the nucleus, NRF2 mediates the upregulation of antioxidant and cytoprotective genes by binding to AREs [68,69,70]. Constitutive activation of NRF2 leads to oncogenesis via activation of Myc, KRAS, PI3K, and BRAF oncogenes [69]. Moreover, NRF2-regulated production of antioxidants, such as glutathione reductase, glutathione S-transferase, and glutathione and glutathione peroxidase, confers chemoresistance to cancer cells [71] against anticancer drugs, including cisplatin via thiol-cisplatin adduct-mediated inactivation of cisplatin [72]. Apart from cell proliferation, NRF2 also mediates cellular motility and invasion by dysregulating the mediator of cellular contractility RhoA/Rho-associated coiled-coil-forming kinase (ROCK1) signaling [73]. Mutations resulting in gain of function of NRF2 [26,74] and loss of functions of KEAP1 resulting in higher levels of free NRF2 have been witnessed in NSCLC and mediate metastasis [74,75] and metabolic remodeling in lung cancer cells [76].

### 3.5. PD-1/PD-L1 Pathway

The programmed cell death protein-1 (PD-1)/programmed cell death ligand-1 (PD-L1) signaling pathway in lung cancer cells is a form of immunosuppressive signaling which helps to null the T cell response against lung cancer cells [77]. The PD-1 receptor and its ligand, PD-L1, are transmembrane proteins expressed on the surface of immune cells, such as T cells, B cells, and antigen-presenting cells [78,79]. PD-L1 is frequently expressed on the surface of malignant cells. Physiological binding of PD-L1 to PD-1 on an activated T cell results in phosphorylative activation of the PD-1, which causes suppression of PI3/Akt signaling in T cells [80]. This inactivation of the PI3K/Akt signaling pathway further downregulates prosurvival proteins, such as Bcl-xL, and results in apoptosis of T cells, thus sparing cancer cells from immune intervention [81].

## 4. Potential Therapeutic Targets for Lung Cancer

A surge of potential therapeutic targets of lung cancer such as ALK, EGFR, BRAF, c-Ros oncogene 1 (ROS1) [82], MET, and RET came to light in the last decade [83]. EGFR is a receptor tyrosine kinase which possesses an extracellular ligand-binding domain [84,85], with its major ligands being epidermal growth factor and transforming growth factor alpha [84], and a cytoplasmic domain rich in tyrosine residues. These two domains are linked by a transmembrane scaffold. Upon ligand-mediated stimulation, EGFR undergoes dimerization and a conformational change conferring autophosphorylation of its intracellular tyrosine residues. This triggers signal transduction through the RAS/RAF/MEK/ERK pathway, or PI3K/Akt/mTOR pathway, or through stimulation of STAT [86,87] which is implicated in cell proliferation and inhibition of apoptosis [86]. After discovering the link between NSCLC and mutations in EGFR, small molecule tyrosine kinase inhibitors were developed and are currently being utilized for targeted therapy in EGFR mutant-positive cases [84]. The most frequently encountered EGFR mutations in NSCLC include substitution of leucine for arginine at codon 858 of exon 21 (L858R), as well as exon 19 deletions [87,88]. A wide repertoire of receptor, signaling, and effector proteins in these pathways serve as potential targets in the treatment of lung cancer [89].

Yet another valuable target for lung cancer is ALK. Like EGFR, ALK is also a tyrosine kinase receptor belonging to the insulin receptor family [90]. In NSCLC, ALK is basically present as a fusion kinase, with ALK being the cytoplasmic domain of the transmembrane receptor rich in tyrosine residues. The extracellular domain consists of the echinoderm microtubule-associated protein-like 4 (EML4) [91,92,93]. Even without stimulation by ligands, this fusion complex undergoes dimerization and autophosphorylation in a manner identical to receptor-bound EGFR. This phosphorylation activation then triggers a signaling cascade through either the RAS/RAF/MEK/ERK pathway, PI3K/Akt/mTOR pathway, or JAK-STAT pathways, or by phospholipase C-γ stimulation which mediates ALK-triggered cell proliferation and survival [90]. Similar ligand-independent signaling in NSCLC tumorigenesis has been witnessed with RET mutations, another tyrosine kinase receptor and novel target for NSCLC treatment. RET is reported to be associated with chimeric kinase protein resulting in an auto-triggering fusion complex, alike to that witnessed with ALK in NSCLC [94].

Another potential target for lung cancer treatment is MET. This oncogene encodes for receptor tyrosine kinase (RTK), which triggers a multitude of signaling cascades, including the PI3K/Akt/mTOR pathway, MAPK/ERK pathway, and JAK/STAT transduction pathway. Although these signaling cascades require ligand-mediated stimulation of the RTK receptor under normal conditions, activating mutations such as METex-14 abolish this prerequisite [82]. Accordingly, MET inhibitors are currently being explored as targeted therapies mitigating lung cancer.

In addition to signaling molecules, microRNAs are emerging as mediators of either tumorigenesis or tumor suppression. They serve as crucial biomarkers as well as therapeutic targets for lung cancers [95]. Certain microRNAs, like microRNA-148a-3p, microRNA-129-5p, and microRNA-218-5p, have been shown to be associated with the radiosensitivity of NSCLC cells. MicroRNA-148a-3p has emerged as a crucial target in catering to patients with radiation-resistant NSCLC; upregulation of microRNA-148a-3p is proposed to enhance radiosensitivity [96]. Also, microRNA-148a-3p acts as a tumor suppressor. It inhibits salt overly sensitive2 (SOS2) and thus prevents the activation of RAS, further obstructing tumor progression [95].

## 5. Current Therapeutic Strategies for Mitigating Lung Cancer and Associated Adversities

The choice of treatment for NSCLC is surgical resection in patients without comorbidities or radiation therapy in patients with existing comorbidities [97]. Additionally, surgery is limited to early-stage lung cancer patients and the majority of the advanced cases are treated either with chemotherapy or radiotherapy or concurrent chemo- and radiotherapy. Radiation therapy, however, is damaging to non-cancerous cells in the vicinity, which may result in side effects like esophagitis, pneumonitis [98], and compromised lung functionality [99]. Surgical options like pneumonectomy are associated with postoperative pulmonary hypertension, acute respiratory distress syndrome (ARDS), and mortality, which was reported to be 8.5% amongst the 294 patients of malignant lung cancer subjected to pneumonectomy in a study carried out by Daffrè and colleagues [100]. For both limited and extensive SCLC, platinum-based combination chemotherapy is the first line of treatment [97,101], although lobectomy and radiotherapy are also employed in mitigating limited SCLC [102]. However, given the associated risks and limited response, benefits of surgical resection in the management of SCLC are subject to debate [101].

A multitude of chemotherapeutic agents are available for treating lung cancer (Table 1). The drug combinations cisplatin and paclitaxel, cisplatin and docetaxel, cisplatin and gemcitabine, and carboplatin and paclitaxel are commonly employed for the treatment of NSCLC. Each of these combinations has similar efficacy against NSCLC and is proven to enhance life expectancy by as much as 1 to 2 years. The combination carboplatin and paclitaxel, however, is generally preferred over other options due to its relatively low toxicity [103]. For SCLC, etoposide in combination with either carboplatin or cisplatin is preferred [104].

Chemotherapy with platinum coordination complexes, such as cisplatin, has been proven to be efficacious in completely resected and excision repair cross-complementation group 1 (ERCC1) protein-negative NSCLC [144]. ERCC1 is a major player involved in repairing cisplatin–DNA complexes [145]. A prolonged survival rate was witnessed when cisplatin plus gemcitabine was given in ERCC1-negative advanced NSCLC. As mentioned above, platinum coordination complexes such as carboplatin and cisplatin, as well as etoposide, are mainstay treatments of SCLC [146]. These compounds have been shown to be highly efficacious in mitigating limited SCLC when combined with thoracic radiation. Despite being first-line treatment against SCLC, a high relapse rate and high overall mortality are seen [147]. Apart from this, all platinum-based anticancer agents pose a risk of causing adverse effects ranging from anorexia, vomiting, diarrhea, alopecia, and stomatitis to ototoxicity [110,148], as well as hepatotoxicity, nephrotoxicity, cardiotoxicity, neurotoxicity, myelosuppression [149], and anaphylaxis [150].

Bevacizumab, a recombinant humanized monoclonal antibody targeting vascular endothelial growth factor (VEGF), has shown promising outcomes in the survival of patients with non-squamous NSCLC when given alone or in combination with platinum-based chemotherapy [151,152]. Bevacizumab is associated with increased risk of developing hypertension, pulmonary hemorrhage, and thromboembolism [35,130]. Cetuximab, a chimeric monoclonal antibody against EGFR, not only blocks EGFR–ligand interaction, but also promotes the internalization and degradation of EGFR, resulting in its downregulation [141]. It is employed for the treatment of advanced NSCLC. Yet another category of drugs that target EGFR include gefitinib, erlotinib, and rociletinib. These drugs act by inhibiting EGFR tyrosine kinase [85]. All agents within this class of EGFR-targeting drugs have been reported to put patients at risk of skin rashes and diarrhea [141,153]. Gefitinib is also known to cause appetite loss, anemia, and sensory neuropathy [154], whereas rociletinib is prone to cause QT prolongation and hyperglycemia [139].

Targeted therapies for mitigating mutation-associated malignancies have been introduced lately. Concurrent administration of dabrafenib (150 mg, twice daily, orally) and trametinib (2 mg, daily, orally) have been approved by the United States Food and Drug Administration (FDA) for treating BRAF V600E mutation-positive metastatic NSCLC. An open-label, multicenter, and multicohort trial of 93 patients found this combination to be effective, but not free of adverse effects. Dabrafenib–trametinib combination therapy has been reported to cause vomiting, diarrhea, pyrexia, dyspnea, edema, and hemorrhage [112]. It is also associated with an increase in alanine aminotransferase and aspartate aminotransferase, as witnessed in phase II trials [155]. In addition to trametinib, other MEK1/2 inhibitors are undergoing trials for the treatment of lung cancer. One of these compounds is selumetinib (75 mg, twice daily, orally), which was reported to be efficacious in treating *KRAS* mutation-positive advanced NSCLC when paired with docetaxel but failed to promote survival in lung cancer patients in phase III trials [119,156].

Sotorasib recently received FDA approval in May 2021 for use in *KRAS* G12C mutation-associated NSCLC [157]. It has been reported to be clinically effective and safe at a tested dose of 960 mg, daily in phase II trials carried out on 126 patients with KRAS G12C mutation-positive NSCLC [158]. Atezolizumab, a monoclonal antibody against programmed death ligand-1 (PD-L1), has shown highly promising outcomes in enhancing survival rates in patients with metastatic lung cancer with EGFR or ALK aberrations and PD-L1 expression, as well as in resected patients diagnosed with stage II or IIIA NSCLC [114]. Similar survival outcomes have also been witnessed for pembrolizumab, yet another approved monoclonal antibody against PD-L1 [116]. Adversities encountered with atezolizumab and pembrolizumab range from pruritis, cough, pyrexia, and gastrointestinal disturbances like vomiting and diarrhea to peripheral edema, dyspnea, and anemia [116,159].

ALK inhibitors like alectinib, brigatinib, ceritinib, and lorlatinib are first-line therapies in advanced ALK-positive NSCLC [160]. Crizotinib is another an inhibitor of ALK phosphorylation which also inhibits c-MET [123] and ROS1 [122]. Crizotinib has received “breakthrough” designation for its remarkable outcomes in NSCLC with METex-14 mutations [161] but has also been reported to cause serious visual impairment [162], hepatotoxicity [163], bradycardia [164], hypogonadism in males [165], and gastrointestinal disturbances [123].

Other treatment options for lung cancer exist, including chemotherapeutic and radiotherapeutic options. A STAT3 inhibitor, napabucasin, has been shown to exhibit synergism with paclitaxel in promoting tumor regression and patient survival in extensively pretreated NSCLC. Reported side effect include diarrhea and hyponatremia [142]. Lung cancer is often treated using a combination of external beam radiation therapy with adjuvant chemotherapy. However, brachytherapy has emerged as a better therapeutic strategy compared to external beam radiation due to its ability to deliver a higher dose of radiation with greater precision, thereby sparing more healthy tissue [166,167].

## 6. Literature Search and Selection Process

**Figure 3:**
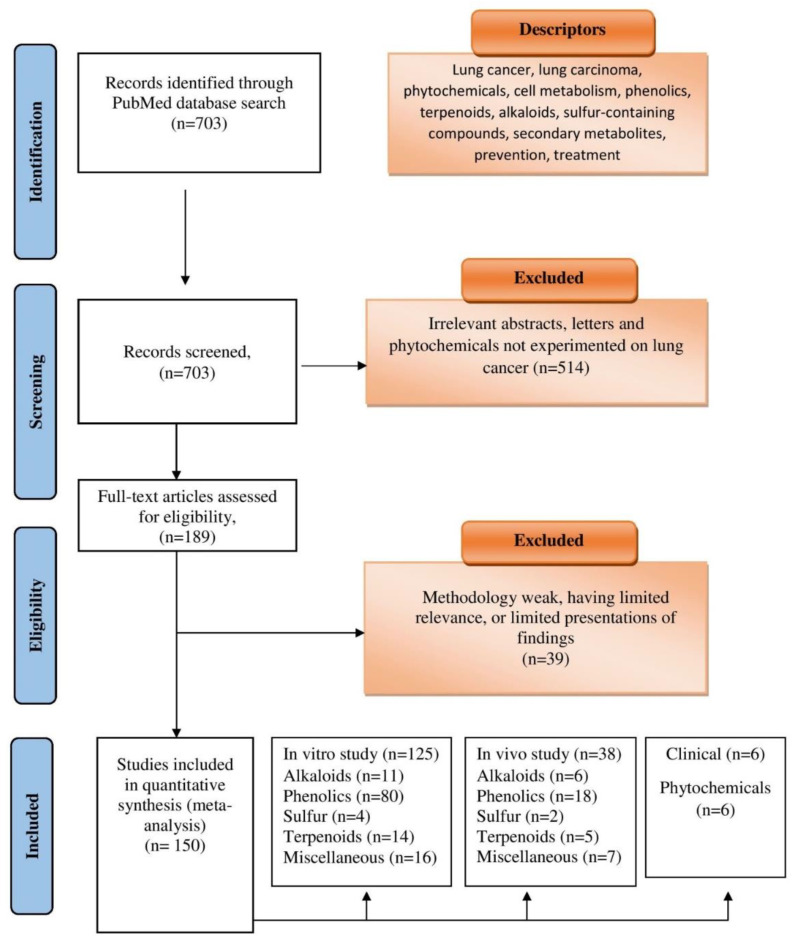
PRISMA flowchart detailing the selection process for included studies. The total quantity of in vitro, in vivo, and clinical experiments (169) is larger than the number of unique studies included in this systematic analysis (150) because several publications contain more than one type of experiment.

The Preferred Reporting Items for Systematic Reviews and Meta-Analyses (PRISMA) criteria [168,169] for methodical reviews were employed when evaluating literature. Searches were conducted in PubMed using keywords and phrases to identify relevant articles. Search criteria consisted of combinations of the following words and phrases: lung cancer; lung carcinoma; phytochemicals; cell metabolism; phenolics; terpenoids; alkaloids; sulfur-containing compounds; secondary metabolites; prevention; treatment. Following identification, articles were evaluated for their content. Reviews and letters, articles not in English, and those concerning phytochemicals outside the context of lung cancer were excluded. Additionally, weak methodology articles and those with little relevance or limited presentation of findings were excluded. A breakdown of the article selection process according to the PRISMA guidelines is represented in Figure 3.

## 7. Anticancer Potential of Bioactive Phytocompounds in Lung Cancer

### 7.1. Preclinical Studies

Plant secondary metabolites are considered safe and effective in the treatment of cancer [170] and act primarily by modulation of numerous signaling pathways [171] Phytochemicals may be classified into four broad groups, such as alkaloids, phenolics, sulfur-containing compounds, and terpenoids. In the recent past, various in vitro and in vivo studies were conducted to elucidate the role of plant phytocompounds in the context lung cancer which are presented in the following subsections.

#### 7.1.1. Alkaloids

Alkaloids represent a large group of organic compounds distinguished by their nitrogen-containing structures. They are present in a wide variety of plants and are known for their cancer preventive and anticancer activities [172]. Their wide-ranging biological activities have prompted extensive research into their potential therapeutic applications in drug discovery and development [173].

##### Acutiaporberine

**Figure 4:**
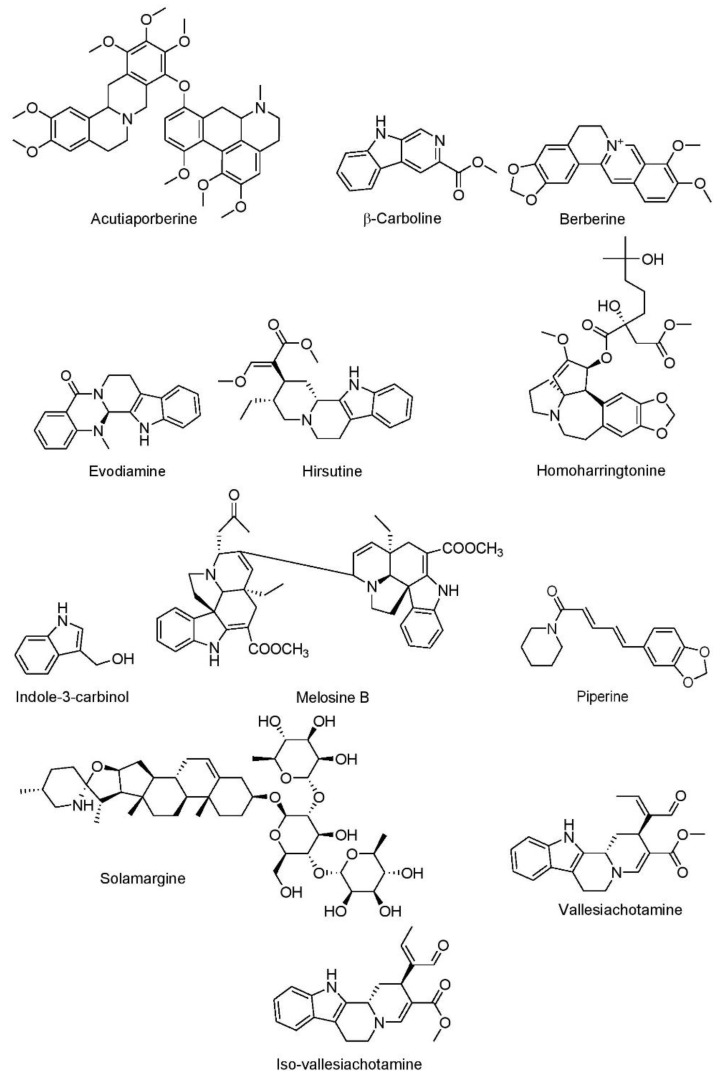
Chemical structures of alkaloids with anticancer activity in lung cancer.

Acutiaporberine (Figure 4) is a bisalkaloid derived from *Thalictrum acutifolium*. Very limited research has been conducted on acutiaporberine since its discovery approximately 20 years ago. In one such study, researchers determined that application of acutiaporberine to 95-D lung cancer cells resulted in upregulation of apoptosis (Table 2). The researchers reported these effects to be mediated by enhancing the Bak/Bcl-2 ratio [174].

##### β-Carboline

β-Carboline is an indole alkaloid (Figure 4) derived from *Peganum harmala* and *Nicotiana tobacum* and has been reported to be effective in the treatment of neurodegenerative diseases, such as Parkinson’s and Alzheimer’s [295]. Investigators observed the role of β-carboline in A549 lung cancer cells and reported cytotoxic effects at a concentration of 1.80 μM. Researchers attributed these anticancer effects to an increase in the expression of ERK1/2 and Akt/mTOR signaling pathways [175].

##### Berberine

Berberine (Figure 4) is a quaternary alkaloid derived from various plants, including Oregon grape, European barberry, goldenseal, phellodendron, goldthread, and tree turmeric, which is reported to inhibit the cell cycle and induce apoptosis in cancer cells [296,297]. Berberine exerts its anticancer activity against A549 and H1299 lung cancer cell lines by promoting Bcl-2-associated X protein (Bax) and caspase-3 Bcl-2-associated X protein (Bax) and caspase-3-mediated apoptosis [176]. In vivo study furthered these results by demonstrating a reduction of Bcl-2 expression and enhanced the Bax- and caspase-3-mediated apoptosis of cancerous cells in the xenografts of athymic nude mice [176].

##### Evodiamine

Evodiamine (Figure 4) is a quinolone alkaloid derived from tetradium fruit that is commonly used for its weight management, analgesic, and neuroprotective effects [298,299,300]. Documented anticancer effects of evodiamine include its ability to inhibit various signaling molecules, such as mTOR, NF-κB, PI3K/Akt, and JAK-STAT [301]. In vivo analysis utilizing nude mice with Lewis lung carcinoma xenografts demonstrated evodiamine’s ability to improve host immune response against tumor cells at doses of 10, 20, and 30 mg/kg (Table 3). Mechanistically, evodiamine was found to reduce the transmembrane glycoprotein mucin 1-C (MUC-1)/PD-L1 expression and increase CD8+ T cells [302].

##### Hirsutine

Hirsutine (Figure 4), an indole alkaloid, is derived from the bark of Uncaria and has been shown to possess antiviral and neuroprotective properties [313]. Hirsutine has proven efficacy against various cancer models, including T cell leukemia and lung cancer [314,315]. In an in vivo lung metastasis model using female BALB/c mice, hirsutine was found to reduce cell invasion and metastasis at an intravenous dose of 25 µM by targeting the NF-κB signaling pathway [303].

##### Homoharringtonine

Homoharringtonine (Figure 4) is an ester of the cephalotaxine alkaloid that is present in *Cephalotaxus harringtonia* belonging to the family Cephalotaxaceae and has displayed antiviral properties [316] and breast anticancer activity by suppression of the miR-18a-3p/Akt/mTOR signaling pathway [317]. In lung cancer, specifically, homoharringtonine has been reported to decrease JAK1/STAT3 signaling at a concentration of 2–4 μM in A549 cells [177]. In another study, at a concentration of 2 µM, homoharringtonine inhibited the expression of KRAS, ERK, Akt, STAT3, CDK4, and CDK6 in A549 and H1299 lung cancer cells. Somewhat paradoxically, researchers also noted a decrease in the expression of tumor suppressor protein p21 and RB-treated cells [178]. These findings are, in part, supported by in vivo experiments. Utilizing a xenograft tumor mouse model, Cao et al. [177] demonstrated the ability of 10 mg/kg homoharringtonine to reduce the cell proliferation by suppressing IL-6/JAK1/STAT3 signaling. Later, Weng et al. [178] also conducted an experiment investigated the effects of 2.5 mg/kg homoharringtonine on mice bearing KRAS mutation-positive xenograft tumors. The researchers determined that treatment with homoharringtonine elevated the expression of caspase-3 and caspase-9 and downregulated Bcl-2 expression.

##### Indole-3-Carbinol

Indole-3-carbinol (Figure 4) is an indole alkaloid and glucosinolate derivative, derived from broccoli, cauliflower, cabbage, and other cruciferous vegetables. In vitro analyses have been conducted to evaluate the anticancer effects of indole-3-carbinol. Dadashpour and Emami [318] report the ability of indole-3-carbinol to cause G1/S cell cycle arrest and apoptosis in several cancer models. More specifically, indole-3-carbinol increased oxidative stress and expression of caspase-3, caspase-7, and caspase-9 to ultimately induce apoptosis in H1299 lung cancer cells at a concentration of 400 μM [179].

##### Melosine B

Melosine B (Figure 4), an indole alkaloid derived from *Melodinus cochinchinensis*, has been demonstrated to be cytotoxic to various cancer cell lines [319]. Researchers report the ability of melosine B to induce apoptosis in A549 lung cancer cells at concentrations of 0.064, 0.32, 1.6, 8, and 40 µM. The investigators did not report any mechanism of action to explain these findings [180].

##### Piperine

Piperine (Figure 4) is an amide alkaloid, commonly found in black pepper. It has several biological activities, such as hepatoprotective, immunomodulatory, antioxidant, antitumor, antidiabetic, and cardioprotective effects [320]. Piperine was reported to induce cell cycle arrest in the A549 lung cancer cell line at a concentration of 50, 100, and 200 μg/mL by upregulating caspase-3 and caspase-9 cascades and the Bax/Bcl-2 ratio [181]. In another in vitro analysis, piperine demonstrated the ability to suppress A549 cell viability and migration at a concentration of 20, 40, 80, 160, and 320 µM by inhibiting ERK 1/2, SMAD 2 phosphorylation, and the transforming growth factor-β (TGF-β) signaling pathway [182].

##### Solamargine

Solamargine (Figure 4) is a steroidal alkaloid and cytotoxic compound derived from *Solanum incanum*, a member of the Solanaceae family, and acts by disrupting the growth of cancer cells [321]. Solamargine was found to inhibit the actions of prostaglandin E2 (PGE2), restrict DNA protein expression, and enhance ERK1/2 phosphorylation in H1650, H1975, PC9, A549, and H1299 lung cancer cell lines at a concentration of 2, 4, and 6 µM [183]. In a xenograft mouse model, solamargine again decreased the PGE2 and DNA protein expression at doses of 4 and 8 mg/kg [183].

##### Vallesiachotamine and Iso-Vallesiachotamine

Vallesiachotamine and iso-vallesiachotamine (Figure 4) are indole alkaloids derived from *Anthocephalus cadamba* which act by promoting apoptosis in cancer cells [322]. In vitro analysis performed by Mishra et al. [184] demonstrated efficacy against the lung cancer cell line H1299 at concentrations of 12.5, 25, 50, 100, and 200 μM. The researchers attribute these anticancer effects to an increase in DNA damage and upregulation of apoptosis.

#### 7.1.2. Phenolics

Phenolics are a diverse group of natural compounds widely distributed in plants. These compounds are characterized by their inclusion of at least one aromatic ring within their chemical structure, which contributes to their antioxidant properties. Plant-derived phenolics play essential roles in numerous biological processes, including defense against pathogens, UV protection, and cell signaling. Based on structural differences, phenolics may be subcategorized primarily into flavonoids and non-flavonoids. Flavonoids are further divided into six major groups: anthocyanidins, flavanols, flavanones, flavones, flavonols, and isoflavonoids. The non-flavonoids may be further subdivided into phenolic acids, stilbenes, and lignans, amongst others. These bioactive compounds have garnered significant interest due to their broad-spectrum biological and pharmacological activities, such as antioxidant, anti-inflammatory, and antineoplastic properties [323,324,325].

##### Acacetin

**Figure 5:**
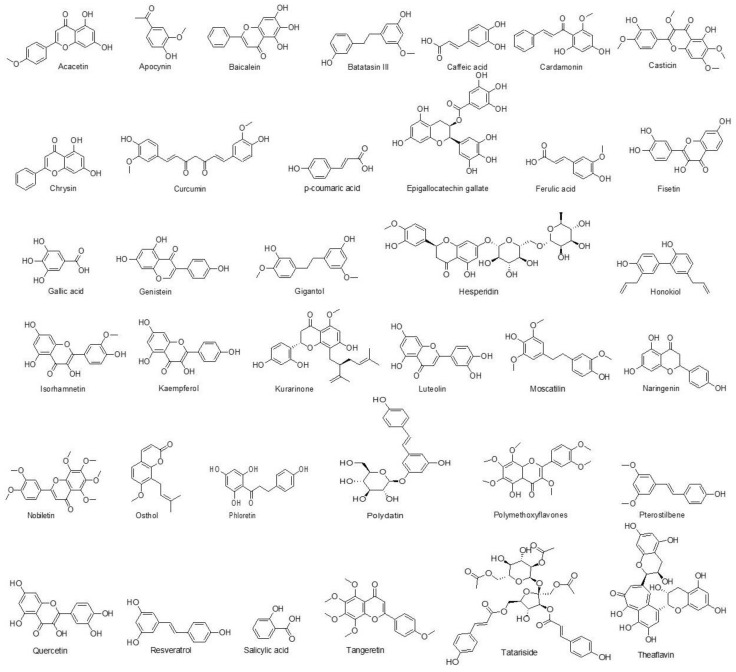
Chemical structures of phenolic compounds with anticancer activity in lung cancer.

Acacetin (Figure 5) is a flavonoid derived from *Tunera diffusa*, *Dracocephalum moldavica* propolis, *Betula pendula*, *Flos chrysanthemi* indici, *Robinia pseudoacacia* chrysanthemum, Calamintha, safflower, and Linaria species. Generally, anticancer effects of acacetin include inhibition of tumor cell migration and invasion [326]. In lung cancer specifically, acacetin has been shown to inhibit cell viability, invasion, migration, and inflammation and accelerate apoptosis in A549 NSCLC cells. Researchers report these effects to be mediated by suppression of the p38α MAPK signaling pathway [185].

##### Apocynin

Apocynin (4-hydroxy-3-methoxy-acetophenone (Figure 5), a phenolic compound derived from *Apocynum cannabinum* and *Picrorhiza kurroa*, has been reported to possess anti-inflammatory, antioxidant, and anticancer effects [327]. Paul et al. [186] investigated the role of apocynin against A549 lung cancer cells and observed decrease in the microtubule network of cells, as well as reductions in proliferation, colony formation, and cell invasion. The same group of investigators further studied these effects by conducting an in vivo study using BALB/c mice with xenograft tumors. They found that treatment with apocynin at doses of 50 and 100 mg/kg inhibited cell growth, invasion, colony formation, and microtubule network. They attribute these findings to cellular microtubule depolymerization, resulting in tumor cell apoptosis.

##### Baicalein

Baicalein (5,6,7-trihydroxyflavone, Figure 5) is a flavonoid derived from the root of *Scutellaria baicalensis* that is known to modulate cardiovascular health [328]. Biacalein has displayed broad anticancer effects by initiating apoptosis via mitochondria and receptor-mediated pathways [329]. In lung cancer cell lines A549 and H1299, baicalein has been shown to suppress the cytoskeleton linker protein ezrin, resulting in decreased cell invasion and metastasis [187]. Researchers validated these findings in an in vivo experiment utilizing BALB/c nude mice with tumor xenografts. Baicalein was also found to inhibit cancer invasion and metastasis at doses of 2.5, 10, and 40 mg/kg [187].

##### Batatasin

Batatasin III (Figure 5) is a stilbenoid compound derived from *Dendrobium draconi*, *Bulbophyllum reptans*, and *Cymbidium aloifolium*. It has numerous pharmacological actions, including antioxidant, anticancer, anti-inflammatory, antidiabetic, antiapoptotic, anticholinesterase, antioxidant, antiherpetic, and antimalarial activities [330]. In vitro analysis of batatasin against H460 lung cancer cells demonstrated its ability to decrease cell proliferation, invasion, and metastasis. Researchers attribute these findings to a decrease in epithelial–mesenchymal transition (EMT) through downregulation of N-cadherin and vimentin and the Akt pathway and upregulation of E-cadherin [188].

##### Caffeic Acid

Caffeic acid (Figure 5) is a polyphenol derived from coffee beans, olives, fruits, carrots, potatoes, and propolis. In hepatocellular carcinoma, caffeic acid has been demonstrated to increase reactive oxygen species and DNA oxidation, as well as decrease angiogenesis [331]. In A549 lung cancer cells, caffeic acid was shown to reduce cell proliferation, adhesion, and migration by inhibition of superoxide production [189].

##### Cardamonin

Cardamonin (Figure 5) is a chalcone compound that is present in cardamom, a spice belonging to the Zingiberaceae family. Anticancer effects displayed by cardamonin include inhibition of the PI3K/Akt pathway in various cancer models [190]. In A549 and H460 lung cancer cells, cardamonin was reported to inhibit the PI3K/Akt pathway and increase the expression of caspase-3, Bcl-2, and Bax at a concentration of 40 μM [190]. In another study, at a concentration of 30 μM, cardamonin inhibited DNA synthesis and promoted apoptosis in A549 cells [191]. Cardamonin analogs, including 4,40-dihydroxylchalcone (DHC) and 4,40-dihydroxy-20-methoxychalcone (DHMC), reduce cell growth and expression of NF-κB at concentrations of 0.445 µM and 0.166 µM, respectively, in A549 and NCI-H460 cell lines [192]. In vivo analysis of the effects of cardamonin demonstrated its ability to inhibit cell proliferation, migration, and angiogenesis in nude mice with xenograft tumors of lung origin. Researchers noted an increase in caspase-3 and Bax expression, with concurrent decreases in the expression of Bcl-2, cyclin D1, CDK4, and PI3K and Akt and mTOR signaling [304].

##### Casticin

Casticin (Figure 5) is a methoxylated flavonol derived from *Vites trifolia*, *Vites agnus-castus*, and *Vites negundo*. Casticin has been demonstrated to possess antiproliferative and proapoptotic effects in numerous types of cancer, including breast, colon, liver, and others [332]. These anticancer effects have been validated in the setting of lung cancer using A549 cells. Researchers demonstrated the ability of casticin to decrease inflammatory mediators such as IL-6, cyclooxygenase-2 (COX-2), and NF-κB, in addition to downregulating chemokine gene expression [193,194].

##### Chrysin

Chrysin (5,7-dihydroxyflavone) (Figure 5) is present in propolis, honey, *Passiflora caerulea*, *Passiflora incarnata*, *Alpinia galangal*, and *Oroxylum indicum* and has been reported to exert its anticancer effects by caspase activation and inactivation of Akt signaling [333]. In A549 lung cancer cells, chrysin has been shown to increase apoptosis by elevating caspase-3, Bcl-2, and Bax expression. Investigators also noted decreased cell proliferation following the application of chrysin. In a follow-up in vivo study, researchers treated tumor-bearing BALB/c mice with chrysin at a dose of 1.3 mg/kg and observed an increase in caspase-3-mediated apoptosis [195].

##### Curcumin

Curcumin (Figure 5) is a spice derived from *Curcuma longa* L. with substantial evidence for its use as a chemotherapeutic agent. Some of the well-established anticancer applications of curcumin include its ability to induce apoptosis and inhibit proliferation of tumor cells by numerous cellular signaling pathways [334]. In A549 lung cancer cells, 10 µM of radiosensitized curcumin was shown to reduce the migration and invasion of tumor cells, likely by suppression of antiapoptotic factors [196]. In another study utilizing A549 cells, curcumin at 10–50 µM increased caspase-3-induced apoptosis by promoting G2/M phase cell cycle arrest and DNA damage and caused stress to the endoplasmic reticulum, resulting in activation of the unfolded protein response and eventual apoptosis in tumor cells [197]. Additional in vitro analysis using HCI-H460 cells also showed that, at a concentration of 30 μM, curcumin induced apoptosis by reducing expression of CDK1 and upregulating caspase-3 and caspase-8 [198]. Moreover, 1–20 μM of curcumin was documented to increase activator protein-1 in the CL1–5 cell lines, thereby reducing cancer cell invasion and metastasis [199]. Curcumin was also shown to cause DNA damage and promote apoptosis in PC-9 cells at a concentration of 50 μM. Researchers attribute these findings to curcumin’s role in reducing the expression of Bcl-2 and cyclin D1 and CDK2, CDK4, and CDK6 gene expression [200]. Furthermore, 5–40 μM of curcumin contributed to apoptosis in NCI-H292 cells with measured reductions in the expression of CDK1 and enhanced expression of caspase-3 and caspase-8 [201].

Limited in vivo experimentation has been conducted with curcumin in the context of lung cancer. Sak [196] utilized a C57BL/6J lung carcinoma mouse model to demonstrate that, at a dose of 100 mg/kg, curcumin effectively reduced angiogenesis and cancer cell proliferation and initiated apoptosis by suppression of prosurvival factors.

##### p-Coumaric Acid

p-Coumaric acid (Figure 5), a hydroxycinnamic acid, belongs to a class of polyphenols found in various edible plants such as tomatoes, carrots, and cereals. It has several biological activities, including anti-inflammatory, analgesic, antioxidant, and antimicrobial properties [335]. p-Coumaric acid was reported to increase apoptosis in A549, NCI-H1299, and HCC827 lung cancer cell lines at a concentration of 10–100 µg/mL by upregulating caspase-3 and caspase-9 [202]. Separate in vitro analysis by Jeong et al. [203] demonstrated the ability of p-coumaric acid to suppress H1993 cell viability at a concentration of 50–100 μM by overcoming the resistance of epidermal growth factor receptor tyrosine kinase inhibitor. Despite promising in vitro studies, the anticancer effect of p-coumaric acid has been relatively understudied in animal models of lung cancer. One available report further describes the proapoptotic effects of p-coumaric acid against a xenograft lung cancer model in nude mice. Researchers found that, at a dose of 50 mg/kg, p-coumaric acid enhanced caspase-3- and caspase-9-mediated apoptosis in xenograft tumors [202].

##### Epigallocatechin Gallate

Epigallocatechin gallate (EGCG, Figure 5), a green tea-derived polyphenol, has been reported to be effective against several types of cancers, including kidney, colon, lung, brain, and breast, as well as in leukemia [336,337]. In A549 and H1299 lung cancer cells, 20–300 μM of EGCG was shown to decrease the tumor cell proliferation likely by suppression of the NF-κB signaling pathway [204]. In another study, at a concentration of 10–100 µM, EGCG decreased the A549 cell proliferation by inhibiting the NF-κB signaling pathway and increasing oxidative stress [205]. EGCG was also shown to reduce the tumor cell proliferation at a concentration of 10, 25, 50, and 100 µM. Researchers attributed these findings to EGCG’s role in reducing nicotine-induced Akt, ERK1/2 signaling, hypoxia-inducible factor-1α (HIF-1α), and vascular endothelial growth factor (VEGF) expression [206]. Moreover, 12.5, 25, and 50 μM of EGCG suppressed tumor cell growth, invasion, and migration with measured reductions in the Bax/Bcl-2 ratio [207]. Datta and Sinha [208] investigated the role of EGCG against A549 lung cancer cells and observed inhibition of tumor cell growth by a decrease in the Nrf2 signaling pathway and increase in the oxidative stress in tumor cells at a concentration of 20–300 μM. The same group of investigators further reported that EGCG reduced the etoposide resistance in A549 and NCI-H23 cells at a concentration of 0.05–500 µM [209]. Moreover, 40 µM of EGCG suppressed the growth of H1299, H460, and A549 cells with measured reductions in miR-210 expressions [210]. Later, in vitro analysis using H1299 and A549 cells also showed that EGCG at a concentration of 10, 20, and 40 µM induced apoptosis by reducing expression of the PI3K/Akt signaling pathway [211]. Additionally, the combination of EGCG (30 µM) and luteolin (10 µM) was documented to induce apoptosis, likely by enhancing p53 mitochondrial translocation and DNA damage, in A549 and H460 cells [212]. Furthermore, theaflavins and EGCG at a concentration of 100 µM suppressed the tumor cell proliferation by enhancing expressions of p53 and inhibiting Bcl-2 expression [213].

In vivo analysis of the effects of EGCG demonstrated its ability to inhibit cell proliferation and promote apoptosis in nude mice with xenograft tumors of lung origin. Researchers noted a simultaneous decrease in nicotine-induced Akt and ERK1/2 signaling [305]. Shi et al. (2015) utilized a xenograft BALB/c athymic nude mouse model to demonstrate that pretreatment of animals with EGCG subcutaneously at 100 µM effectively reduced cancer cell proliferation by suppression of nicotine-induced Akt and ERK1/2 signaling [206]. Researchers validated these findings in an in vivo experiment utilizing a nude mouse model with tumor xenografts. EGCG was also found to inhibit cisplatin-induced lung tumorigenesis at doses of 1.62 mg/kg [306].

##### Ferulic Acid

Ferulic acid (Figure 5) is a hydroxycinnamic acid derived from fruits and vegetables such as sweet corn, tomatoes, and rice bran, and has been found to have antioxidant and anticancer activity [338,339]. In an in vitro study carried out utilizing A549 lung cancer cells, 200 µM of ferulic acid decreased cell proliferation, adhesion, and migration. Researchers believe these results to be mediated by inhibition of superoxide production [189].

##### Fisetin

Fisetin (Figure 5) is a flavone present in numerous vegetables and fruit, such as apples, strawberries, grapes, persimmons, cucumbers, and onions. Fisetin is reported to be efficacious in the treatment of numerous malignancies, including breast, cervical, prostate, lung, skin, colon, and gastric cancers, as well as hepatocellular carcinoma, leukemia, and myeloma. Fisetin has displayed anticancer effects by modulating multiple signaling pathways such as Akt/mTOR, Axl, MAPK, PARP, PI3K, and ERK1/2 [340]. In A549 lung cancer cells, fisetin has been shown to induce apoptosis, likely due to its ability to downregulate PI3K/Akt/mTOR signaling [214]. In two studies, Kang et al. [215,216] demonstrated the ability of 75µg/mL of fisetin to decrease cell proliferation in NCI-H460 cells. The investigators believe these results to be mediated by a reduction in the expression of Bcl-2, with increases in the expression of caspase-9 and caspase-3. In a separate study, fisetin was noted to reverse acquired erlotinib resistance of HCC827-ER lung adenocarcinoma cells at concentrations ranging from 10–120 μM. The researchers attributed these findings to fisetin’s role in suppressing the Axl, MAPK, and Akt signaling pathways [217].

##### Gallic Acid

Gallic acid is a phenolic acid (Figure 5), commonly found in bearberry, pomegranate, gallnuts, oak bark, and several other plants, and is well known for its gastrointestinal, cardiovascular, and neuropsychological medicinal properties as well as antioxidant, anti-inflammatory, and antineoplastic properties [341]. In Calu-6 and A549 lung cancer cell lines, gallic acid enhances oxidative stress, decreases the glutathione (GSH) levels, and inhibits cell growth [218]. Researchers attribute these findings to be due to gallic acid’s ability to downregulate STAT3-regulated tumor-promoting gene expression, resulting in cell cycle arrest and apoptosis. Furthermore, gallic acid has been shown to induce cell cycle arrest and upregulate apoptosis in H1975 and H1993 cell lines, likely due to its role in decreasing STAT3 phosphorylation but also due to reduced expression of Bcl-2, cyclin D, NF-κB, and IL-6 [219]. In an in vivo study, gallic acid reduced Src-mediated phosphorylation of STAT3, thereby promoting cell cycle arrest and apoptosis in a mouse xenograft tumor model [219].

##### Genistein

Genistein (Figure 5) is a phytoestrogen primarily derived from legumes and has been proven effective against numerous types of malignancies including liver, breast, prostate, pancreatic, lung, skin, and cervical cancer [342]. In A549 lung cancer cells, genistein has been demonstrated to accelerate trichostatin A-induced caspase-3 activity, thereby causing cell apoptosis [220]. Genistein was also reported to inhibit NF-κB DNA-binding affinity and downregulate expression of COX-2, p-Akt, EGFR, and PGE2, resulting in decreased cell proliferation and upregulation of apoptosis in H3255, H1650, and H1781 lung cancer cell lines [221]. These results have been further validated in two additional studies in which genistein increased apoptosis and cell cycle arrest in the SPC-A-1 line [222] and H460 cell lines [223].

##### Gigantol

Gigantol (Figure 5) is a bibenzyl phenolic compound derived from orchids and is known to have antioxidative, antinociceptive, antispasmodic, anti-inflammatory, and anticancer activity [343]. In lung cancer cell line A549, gigantol has been shown to suppress cell proliferation and enhance apoptosis in tumor cells at a concentration of 25, 50, and 100 µM. Investigators report these effects to be mediated by inhibition of Bcl-2 expression and upregulation of Bax expression and Wnt/β-catenin signaling [224]. In another study, gigantol has been shown to inhibit cell proliferation in H460 lung cancer cells at a concentration of 50 μM. The investigators noted these findings to be associated with inhibition of EMT transcription factor expression [225]. In H460 lung cancer cells, gigantol was shown to destabilize tumor integrity via suppression of the PI3K/Akt/mTOR and JAK/STAT pathways at concentrations of 20–200 µM [226]. To validate these findings, in vivo experimentation has been conducted with lung cancer utilizing a xenograft tumor mouse model. Researchers found that, pretreated at a dose of 20 µM, gigantol reduced cell proliferation by suppressing PI3K/Akt/mTOR and JAK/STAT pathways [226].

##### Hesperidin

Hesperidin (Figure 5) is a flavanone glycoside present in citrus fruits with documented antioxidative, anti-inflammatory, cardiovascular, antiobesity, and anticancer activities [344]. Specifically, hesperidin was reported to promote apoptosis in A549 and NCI-H358 lung cancer cells at concentrations of 5–50 μM. Researchers suggest these results are due to hesperidin’s ability to promote mitochondrial membrane disruption and production of caspase-3, while also enhancing NF-κB signal transduction pathways [227]. A separate study conducted by Jeong et al. [203] demonstrated that, at a concentration of 100 μM, hesperidin reduced cell proliferation and growth in H1993 cell lines. They believe these results to be mediated by overcoming the resistance of EGFR tyrosine kinase inhibitor.

##### Honokiol

Honokiol (Figure 5), a lignan belonging to the genus *Magnolia*, has been revealed to exhibit antiproliferative effects against several cancer cells, including bladder, bone, brain, blood, breast, and colon cancer [345]. When 5, 10, or 20 μM of honokiol was applied to A549 and 95-D lung cancer cell lines, researchers observed inhibited cell proliferation and migration, which they attributed to resultant increases in Bax, caspase-9, and PERK phosphorylation [228,229]. Separate analysis using A549 and LL/2 cell lines further demonstrates the anticancer potential of honokiol as researchers found that application of honokiol to these cell lines promoted apoptosis and regulated vascular endothelial growth factor-A (VEGF-A) expression [230]. In an in vivo analysis, honokiol administered at doses of 7.5, 37.5, and 75 μM/kg led to apoptosis of lung cancer cells in an orthotopic model using NOD/SCID mice. The researchers believed that honokiol exerted anticancer effects by enhancing oxidative stress, mitochondrial Prx3 oxidation, and AMPK pathway activation and inhibition of STAT3 phosphorylation [307].

##### Isorhamnetin

Isorhamnetin (Figure 5), a bioflavonoid that is derived from *Hippophae rhamnoides* L. and *Ginkgo biloba* L., is reported to be efficacious in the management of cerebrovascular and cardiovascular diseases [346]. At a concentration of 16 μM, isorhamnetin was shown to reduce cancer cell proliferation and colony formation and increase apoptosis via caspase activation in A549 cells [233]. In a separate study, 25 μM of isorhamnetin also caused an increase in mitochondrial disruption and caspase-induced apoptosis of A549 cells [234].

##### Kaempferol

Kaempferol (Figure 5) is a flavonol found abundantly in broccoli, yellow fruits, and grapes. In addition to its documented anticancer activities, kaempferol is said to possess neuroprotective, antimicrobial, antioxidant, and anti-inflammatory properties [347]. In lung cancer, 10–140 μM of kaempferol increased expression of EMT-related protein E-cadherin and reduced expression of vimentin, resulting in reduced cell growth and proliferation of A549 cells [235]. In another study utilizing identical cells, kaempferol was shown to decrease cell proliferation, migration, and invasion at a concentration of 25 μM. The researchers noted the role of kaempferol in reducing Akt1-mediated phosphorylation and expression of transforming growth factor-β1 in treated A549 cells [236]. A separate study found that, at concentrations of 30, 50, and 80 μM, kaempferol was able to increase the oxidative stress and caspase-3-induced apoptosis of H460 cells [237].

##### Kurarinone

Kurarinone (Figure 5) is a flavanone derived from *Sophora alopecuroides* and is reported to have immunosuppressive effects and antioxidant activity [348]. When 5.8 µg/mL of kurarinone was applied to H460 cells, investigators observed its ability to cause G2/M blockade and enhance apoptosis. These findings are supported by measurable decreases in NF-κB signaling and tyrosine kinase activity [238]. In another in vitro analysis, researchers reported that kurarinone decreased the expression of EMT-related proteins and MMP-2 in H1688 and H146 cell lines at IC_50_ values of 12.5 and 30.4 µM, respectively, thereby decreasing cell viability, invasion, and migration of tumor cells [239]. One in vivo study of kurarinone demonstrated that a dose of 100 mg/kg increased the rate of apoptosis in BALB/c nude mice with xenografted tumors. Researchers believe these effects to be mediated by an increase in caspase-3 expression [238].

##### Luteolin

Luteolin (Figure 5) is a flavone present in several vegetables and fruits and is reported to have several therapeutic activities, such as antioxidant, antimicrobial, anticancer, neuroprotective, antiviral, cardioprotective, and anti-inflammatory properties [349,350]. Luteolin has been observed to promote apoptosis in A549 cells at a concentration of 20–80 μM. Researchers also noted an increase in G2/M phase cell cycle arrest, Janus kinase (JNK) and Bax expression, procaspase-9 cleavage, and caspase-3 [240]. In another study utilizing A549 cells, 25–100 μM of luteolin decreased cell motility and migration and upregulated apoptosis. The researchers attribute these findings to luteolin’s ability to enhance MEK/ERK signaling and upregulate expression of caspase-3 and caspase-9 [241]. Moreover, additional in vitro analysis supported luteolin’s role as a proapoptic regulator by its action on A549 and H460 cell lines. At a concentration of 10–100 μM, luteolin enhanced miR-34a-5p via targeting MDM4 expression and induced apoptosis in these cancer cell lines [242,243]. Interestingly, luteolin was discovered to downregulate Bad expression in NCI-H460 cells at concentrations of 20–160 μM. Despite this finding, application of luteolin caused increased apoptosis, which was attributable to more significant upregulation of caspase-3 and suppression of Bcl-2 [244].

##### Moscatilin

Moscatilin (Figure 5) is a bibenzyl phenolic compound, derived from stems of the orchid *Dendrobium loddigesii*, which is reported to have antimetastatic properties in hepatocellular carcinoma by targeting the Akt/NF-κB signaling pathway [351]. In the H460 lung cancer cell line, moscatilin was shown to possess antiproliferative properties. Researchers noted that application of moscatlin caused decreased expression of ERK, EMT, Akt, and Cav-1 [245].

##### Naringenin

Naringenin (Figure 5), a flavanone derived from citrus fruits and grapes, has displayed antioxidant, antiviral, antitumor, antibacterial, antiadipogenic, anti-inflammatory, and cardioprotective effects [352]. When applied to A549 cells, naringenin was shown to decrease cell proliferation, invasion, and metastasis via suppression of MMP-2, MMP-9, and the Akt pathway [246]. In another study, naringenin inhibited tumor cell migration and invasion and promoted apoptosis. Researchers again found reduced expression of MMP-2 and MMP-9 but enhanced caspase-3 and p38 MAPK [247].

##### Nobiletin

Nobiletin (Figure 5) is a flavonoid derived from citrus peels that causes tumor cell apoptosis and prevents myocardial injury via the PI3K/Akt signaling pathway [353]. In A549 lung cancer cells, nobiletin decreased the expression of Akt, GSK3β, β-catenin, and multidrug resistance-associated protein (MRP1) expression, while it increased caspase-3-mediated apoptosis and polymerase cleavage [248]. Researchers expanded upon this study by performing in vivo experiments utilizing a xenografted BALB/c nude mouse model. Again, nobiletin was found to enhance caspase-3-mediated apoptosis and DNA polymerase cleavage, with concurrent downregulation of Akt signaling and MRP1 at a dose of 40 mg/kg [248].

##### Osthol

Osthol (Figure 5), a coumarin derivative derived from *Angelica pubescens* and *Cnidium monnieri*, is reported to exert antitumor, neuroprotective, anti-inflammatory, osteogenic, antimicrobial, cardiovascular protective, and antiparasitic effects [354]. In A549 lung cancer cells, osthol was shown to decrease cell proliferation, invasion, and metastasis at concentrations of 25, 50, 100, 150, and 200 μM. Researchers credit these anticancer effects to a reduction in the expression of cyclin B1, p-Cdc2, and Bcl-2, with accompanying inhibition of PI3K/Akt signaling [249]. In another study, 80 µM of osthol decreased cell invasion and migration by reducing the MMP-2 and MMP-9 expression in A549 cells [250,251]. In another in vitro study, osthol decreased TGF-β-induced EMT, NF-κB, and Snail signaling pathways, resulting in decreased cell invasion, migration, and metastasis in A549 cells at 5–80 μM concentrations [252].

##### Phloretin

Phloretin (Figure 5) is a dihydrochalcone derivative present abundantly in strawberries and apples. Reported pharmaceutical applications of phloretin include antioxidant, anticarcinogenic, antidiabetic, and hepatoprotective effects [355]. In lung cancer, 25–75 μg/mL of phloretin was demonstrated to induce apoptosis in several cell lines, including A549, Calu-1, H838, and H520 cells. Researchers report a reduction in Bcl-2, MMP-2, and MMP-9 expression, as well as upregulation of caspase-3 and caspase-9 following treatment [253]. Separate in vitro analysis of phloretin utilizing A549 cells indicated that, at concentrations of 25, 50, 100, or 200 μM, cancer cell migration, invasion, and metastasis were reduced. The investigators noted an increased phosphorylation of p38 MAPK and upregulation of JNK1/2, caspase-3, and caspase-9, with concurrent reductions in the expression of Bcl-2 and NF-κB [254].

##### Polydatin

Polydatin (Figure 5) is a glycosylated form of resveratrol derived from *Polygonum cuspidatum* and is reported to be an efficacious antioxidant and anti-inflammatory agent. Furthermore, polydatin possesses greater capacity to modify the gut microbiota and enhance lipid metabolism in comparison to resveratrol [356]. At a concentration of 50 µM, polydatin was shown to reduce tumor cell proliferation and colony formation via enhancing the Bak/Bcl-2 ratio in A549 and NCI-H1975 cell lines [255].

##### Polymethoxyflavones

Monodemethylated polymethoxyflavones derived from the peels of *Citrus sinensis* have been reported to have numerous therapeutic applications, including anticancer, anti-inflammatory, and antiatherogenic properties [357]. In vitro analysis utilizing H1299 cells demonstrated the ability of polymethoxyflavones to induce apoptosis and regulate cancer cell metabolism. Researchers attribute these effects to a reduction in the expression of iNOS, COX-2, and myeloid leukemia cell differentiation protein (Mcl-1), with increased expression of caspase-3 [231].

##### Pterostilbene

Pterostilbene (Figure 5), a stilbenoid bearing chemical resemblance to resveratrol, is present in *Pterocarpus marsupium* and blueberries. Anticancer applications of pterostilbene are well documented and include discussions of its roles in inhibition of tumor growth, angiogenesis, and metastasis [358]. Application of pterostilbene to NCI-H460 and SK-MES-1 lung cancer cells resulted in increased apoptosis and diminished cell viability, likely due to an upregulation of caspase-3- and caspase-7-induced cell death [256].

##### Quercetin

Quercetin (Figure 5) is a vastly abundant natural flavonoid, present in berries, apples, vegetables, grapes, onions, tomatoes, red wine, and tea [359]. Medicinal applications of quercetin have been researched greatly and include applications as an anti-inflammatory, antioxidant, and anticancer agent, in addition to a regulator of cardiovascular disease [360,361]. When applied to A549 cells, quercetin was shown to reduce cell growth and promote apoptosis. Researchers also noted an increase in the Bc1-2 gene in treated cells [257]. In an in vivo study, quercetin was shown to enhance cancer cell apoptosis in the xenografts of BALB/c nude mice at a dose of 8 mg/kg. The researchers attributed these findings to quercetin’s role in reducing Bcl-2 expression and augmenting Bax gene expression [257].

##### Resveratrol

Resveratrol (Figure 5) is a stilbenoid compound found in grapes, blueberries, plums, apples, and peanuts which has been reported to exhibit antiproliferative activities against various cancer cells and animal tumor models [362,363,364]. In lung cancer, resveratrol inhibits TGF-β1-induced EMT at a concentration of 20 μM in A549 cells, thereby preventing cell invasion and metastasis [258]. Another study determined that resveratrol also initiates caspase-3-mediated apoptosis when applied at a concentration of 8.9 μM to A549 cells [259]. Apoptosis was also observed when a lower concentration of resveratrol (1–10 μM) was applied to the H1993 cell line, presumably by disabling the resistance of EGFR tyrosine kinase inhibitor [203]. Limited in vivo data support the proapoptotic effects of resveratrol. Researchers observed an increase in apoptosis of the xenograft tumor cells of BALB/c nude mice when they were treated with 15, 30, or 60 mg/kg resveratrol, probably due to upregulation of caspase-3 [259].

##### Salicylic Acid

Salicylic acid (Figure 5) is a phenolic compound derived from the bark of the willow tree. It is documented to have anti-inflammatory and analgesic activity, in addition to being used to treat several skin disorders, such as acne, psoriasis, dandruff, seborrheic dermatitis, corns, and warts [365]. When applied to A549 cells, salicylic acid was shown to increase cell cytotoxicity and apoptosis at concentrations from 1.5–9.5 mM. No mechanism of action is suggested by the investigators to explain these findings [260].

##### Tangeretin Derivative

Tangeretin derivative (5-acetyloxy-6,7,8,4′-tetramethoxyflavone, Figure 5) belongs to the class of flavonoids and is present in citrus peels. This compound possesses various therapeutic activities, such as hepatoprotective, antioxidant, antitumor, anti-inflammatory, and neuroprotective effects [366]. With regard to lung cancer, tangeretin derivative has been shown to decrease tumor cell proliferation, metastasis, and angiogenesis in CL1-5, H1299, H226, and A549 cell lines. Researchers believe these effects to be mediated by an increase in G2/M phase arrest and mitochondrial membrane disruption and by inhibition of the PI3K/Akt/mTOR signaling pathway [261]. In an in vivo study, tangeretin derivative also led to disruption of the mitochondrial membrane and suppressed the Akt/mTOR signaling pathway, resulting in decreased cell proliferation, migration, and angiogenesis in the xenograft tumor cells of BALB/c athymic nude mice. Furthermore, the researchers observed an enhancement of caspase-mediated apoptosis at the given dose of 20 mg/kg [261].

##### Tatariside

Tatarisides B, C, and D (Figure 5) are flavonoids derived from the roots of Tartary buckwheat. Collectively, these phytochemicals are reported to have antitumor, anti-inflammatory, antioxidant, antidiabetic, and hepatoprotective activities [367]. Concerning lung cancer, Tatarisides B, C, and D have been shown to increase apoptosis and cell cytotoxicity in A549 cells. However, Tatariside C was found to be most potent against A549 cells [262]. Additional efforts are required to elucidate the mechanistic action of these compounds.

#### 7.1.3. Sulfur-Containing Compounds

Sulfur-containing compounds in plants are a diverse group of chemical substances that contain sulfur atoms within their molecular structures. These compounds are essential for the growth, development, and defense mechanisms of plants. Glutathione, for example, plays a crucial role in cellular detoxification and antioxidant defense in both humans and plants. Another important sulfurous group of natural compounds are the glucosinolates, which contribute to the characteristic flavors and odors of certain plants, such as cruciferous vegetables. The study of these compounds in plants is of considerable interest due to their diverse biological functions and their potential applications in health promotion and disease mitigation [368,369,370,371,372].

##### Allicin

**Figure 6:**
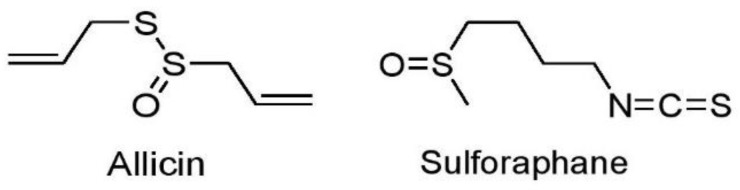
Chemical structures of sulfur compounds with anticancer activity in lung cancer.

Allicin (Figure 6) is an organosulfur compound derived from garlic and has various biological activities such as anthelmintic, antimicrobial, nematocidal, antioxidant, anticancer, and immunomodulatory actions [373]. In an in vitro study, allicin demonstrated effectiveness against cisplatin-resistant A549 and NCI-H460 cells, evidenced by increased ROS-mediated cell death and decreased proliferation. The researchers noted downregulation of cadherin 2 (N-cadherin) and upregulation of cadherin 1 (E-cadherin), with concurrent suppression of hypoxia-inducible factors (HIF-1α and HIF-2α) [263].

##### Sulforaphane

Sulforaphane (Figure 6) is an organosulfur compound, present in cruciferous vegetables such as broccoli and cabbage, and has displayed broad anticancer activity by inhibiting phase I metabolic enzymes and accelerating cell cycle arrest in G2/M and G1 phases, oxidative stress, cell migration, and proliferation [372,374]. Sulforaphane has been shown to decrease the levels of miR-616-5p and GSK3β/β-catenin signaling and to increase S/G2–M phase cell cycle arrest in lung cancer cell lines H1299, 95-C, and 95-D [264]. In another study, sulforaphane downregulated histone deacetylase and enhanced apoptosis by causing cell cycle arrest in G2/M phase in A549 and H1299 [265]. Additional research conducted on sulforaphane demonstrated its ability to act as an epigenetic modulator of miR-21 and decrease CDH1 and DNMT protein levels in A549 lung cancer cells [266]. In an in vivo study, nude mice with xenograft tumors treated with sulforaphane experienced increased apoptosis of tumor cells. The researchers suggest these effects to be a result of downregulated histone deacetylase and promotion of G2/M phase cell cycle arrest [265]. In another study, sulforaphane at a dose of 50 mg/kg was administered to nude mice with xenograft tumors. The researchers observed decreased tumor cell invasion and migration, which they attribute to sulforaphane’s role in enhancing the levels of E-cadherin and ZO-1 and decreasing N-cadherin and Snail 1, thereby causing ERK5 activation [308].

#### 7.1.4. Terpenoids

Terpenoids, also known as isoprenoids, form an extensive and diverse group of compounds found abundantly in natural resources. These compounds are characterized by their structural backbone which is comprised of isoprene units. Terpenoids play crucial roles in various biological processes, including photosynthesis, pigmentation, and defense mechanisms against herbivores and pathogens. Perhaps the most well-known terpenoids include carotenoids, which are responsible for the vibrant colors of fruits and vegetables. Other examples of naturally occurring terpenoids include those present in the essential oils of plants utilized in aromatherapy and traditional medicines. Their historic uses and wide range of purported biological activities have attracted significant interest in pharmaceutical research; in fact, many terpenoids have been shown to exhibit potential therapeutic properties with applications in cancer drug development [375,376,377].

##### Abietane Diterpene

**Figure 7:**
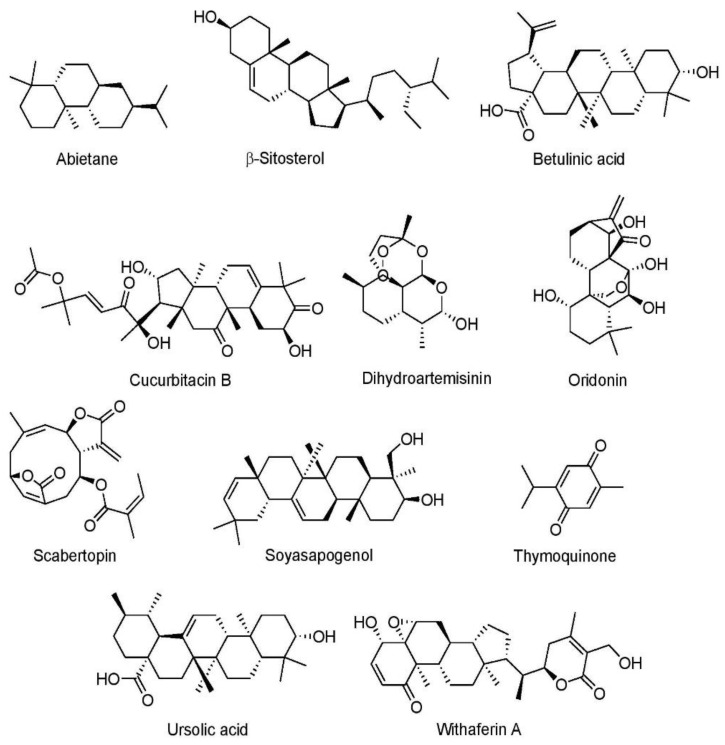
Chemical structures of terpenoids with anticancer activity in lung cancer.

Abietane diterpene (6,7-dehydroroyleanone, Figure 7) is derived from the essential oil of *Plectranthus madagascariensis* and exerts several therapeutic properties, such as antimicrobial, antileishmaniasis, antimalarial, antiviral, antiulcer, antioxidant, and anticancer effects [376]. Garcia et al. [267] demonstrated the ability of abietane to upregulate apoptosis in NCI-H460 and A549 lung cancer cells. The researchers noted an increase in caspase activation and metaphase arrest.

##### β-Sitosterol

β-Sitosterol (Figure 7) is a phytosterol belonging to the class of triterpenoids. Derived from *Grewia tiliaefolia*, β-sitosterol possesses several therapeutic activities, such as antioxidant, antidiabetic, antimicrobial, anticancer, and immunomodulatory effects [377]. β-sitosterol was reported to inhibit the cell cycle at the G2/M phase and initiate apoptosis when applied to A459 cells [268].

##### Betulinic Acid

Betulinic acid (Figure 7) is a pentacyclic triterpenoid primarily derived from the bark of white birch trees and has been shown to promote mitochondrial oxidative stress, regulate transcription factors, and inhibit STAT and activator of the NF-κB signaling pathway in prostate, breast, colorectal, and lung cancers [378]. In an in vivo model of lung cancer, betulinic acid at a dose of 50 or 75 mg/kg was shown to inhibit the migration and proliferation of cancer cells in nude mice bearing xenograft tumors. The researchers noted that betulinic acid acted as an Skp2-SCF E3 ligase inhibitor, thereby inhibiting cancer cell metastasis and proliferation [309].

##### Cucurbitacin B

Cucurbitacin B (Figure 7) is a triterpene obtained from the Cucurbitaceae family which possesses several bioactivities, such as anti-inflammatory, anticancer, and hepatoprotective properties [379]. In lung cancer, cucurbitacin B has been shown to reduce cell proliferation, migration, invasion, and metastasis in A549 cells. Researchers report these findings to be associated with reduced expression of CDK2, CDK4, cyclin D, cyclin E, and mortalin and increased p53 and collaborator of ARF (CARF) proteins [269].

##### Dihydroartemisinin

Dihydroartemisinin (Figure 7) is a semi-synthetic derivative of artemisinin derived from *Artemisia annua* and is reported to have antimalarial, antiviral, anti-inflammatory, and anticancer activity [380]. In the context of lung cancer, dihydroartemisinin is reported to cause apoptosis by enhancing the p38 MAPK expression in the PC-14 cell line at a concentration of 1 μg/mL [270]. In a similar in vitro analysis, researchers reported that dihydroartemisinin increased the expression of p38 MAPK in LLC cells at concentrations of 5, 10, 20, and 40 µg/mL [271]. Another study found that 0.23–749.90 μM of dihydroartemisinin also caused a decrease in transferrin receptor expression in the A549 and H1299 lung cancer cell lines by causing cell cycle arrest in G1 phase [272].

##### Oridonin

Oridonin (Figure 7) is a diterpenoid derived from the Chinese herb *Rabdosia rubescens* and has been reported to have antifibrotic, antibacterial, anti-inflammatory, and anticancer effects [381]. In lung cancer, oridonin decreases cell migration, invasion, and metastasis via mesenchymal transition in the H1975 cell line at a concentration of 10 µM [273]. Additional analysis reported that concentrations of 10, 20, and 30 μM of oridonin increased Bax expression in A549 cells, thereby enhancing cisplatin-induced apoptosis by the AMPK/Akt/mTOR pathway [274].

##### Scabertopin

Scabertopin (Figure 7), a germacrane-type sesquiterpene lactone, is derived from *Elephantopus scaber*. It has displayed an anticancer effect in bladder cancer by modulating ROS and intracellular signaling [382]. In a xenograft mouse model of lung cancer, scabertopin was shown to promote cancer cell death through increased Bax expression and ROS-mediated apoptosis at a dose of 20 mg/kg [310].

##### Soyasapogenol

Soyasapogenol (Figure 7) is a pentacyclic triterpenoid present in soy-based foods and has been shown to reduce the proliferation, migration, and invasion of H1299 cancer cells, with concurrent upregulation of caspase-mediated apoptosis. Researchers also noted a reduction in CDK2, CDK4, cyclin A, and cyclin D1 expression, as well as suppression of pATR-Chk1 signaling [275]. In an in vivo study, 15 mg/kg of soyasapogenol enhanced apoptosis of xenografted tumor cells in a mouse model. Researchers also noted reduced migration and invasion of cancer cells, further confirming soyasapogenol’s role as a potential anticancer pharmaceutical [275].

##### Thymoquinone

Thymoquinone (Figure 7), a monoterpene derived from the seeds of *Nigella sativa*, is well documented for its role in the management and treatment of numerous types of cancers, such as breast and colon cancers, in addition to osteosarcoma [383,384]. The anticancer activities of thymoquinone are primarily exerted through alterations in several oncogenic pathways, such as regulation of oxidative stress, inflammation, metastasis, and angiogenesis [385,386]. In one in vitro study, thymoquinone was found to promote caspase-3-induced apoptosis of LNM3 lung cancer cells. Building from their in vitro analysis, the researchers believed that thymoquinone increased cell death via caspase-3-mediated apoptosis in a xenografted nude mouse model at a dose of 10 mg/kg [276].

##### Ursolic Acid

Ursolic acid (Figure 7) is a pentacyclic terpenoid found abundantly in *Ilex paraguarieni*, *Mimusops caffra*, and *Glechoma hederacea*. In addition to its documented anticancer activities, ursolic acid is said to possess anti-inflammatory, antidiabetic, antibacterial, and antioxidant effects [387,388]. At a concentration of 11, 22, 44, and 88 µM, ursolic acid decreased cell viability and enhanced autophagy in A549 lung cancer cell lines. Researchers observed these changes to be associated with augmented ratio of LC3–phosphatidylethanolamine conjugates (LC3-II/LC3-I) and enhanced expression of ubiquitin-binding protein (p62), PTEN-induced kinase 1 (PINK1), and Nrf2, with reductions in p-Akt and p-mTOR expression [277]. A separate study found that, at a concentration of 0.001–0.1 µM, ursolic acid caused inhibition of tumor cell proliferation and angiogenesis in H1975 cells. Investigators found that application of ursolic acid resulted in decreased N-cadherin and TGF-β1 expression, with enhanced expression of E-cadherin, MMP-2, and MMP-9 [278]. In another study utilizing A549, H460, H1975, H1299, H520, H82, LLC, and H446 cell lines, 5–40 µM of ursolic acid decreased cell proliferation and angiogenesis. The researchers attributed these findings to ursolic acid’s ability to upregulate LC3-II protein and cleaved PARP expression, while downregulating Bcl-2, p-S6K T389, and p-Akt expression [279].

##### Withaferin A

Withaferin A (Figure 7), a terpenoid phytochemical historically used in Ayurvedic medicine, is derived from *Withania somnifera* and has been purported to possess immunomodulatory, antibacterial, and cardioprotective effects [389,390]. General anticancer activities of withaferin A include induction of apoptosis via p53 and suppression the activity of TASK-3 channels [391,392]. When studied in the context of lung cancer, withaferin A was demonstrated to promote apoptosis and increase oxidative stress in A549 lung cancer cells [280].

#### 7.1.5. Miscellaneous Compounds

##### Cannabidiol

**Figure 8:**
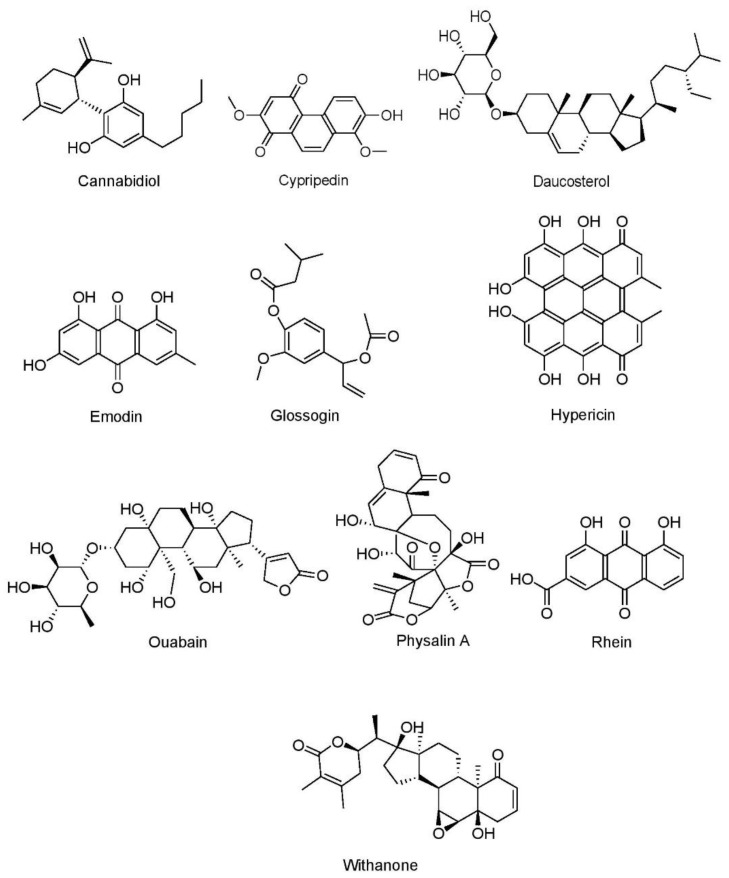
Chemical structures of miscellaneous phytochemicals with anticancer activity in lung cancer.

Cannabidiol (Figure 8) is derived from the plant *Cannabis sativa* and is reported to have several therapeutic benefits, such as anticonvulsant, analgesic, antipsychotic, neuroprotective, anxiolytic, anti-inflammatory, and antioxidant activities [393]. Cannabidiol has been demonstrated to be effective in lung cancer. When 3 µM of cannabidiol was applied to A549 and H460 cells, researchers observed reduced lymphoid trafficking and increased apoptosis [281,282]. In another study, 1–10 µM of cannabidiol was reported to inhibit lymphoid trafficking and enhance COX-2, thereby negatively regulating the growth of lung cancer in A549 and H460 cells [283]. Moreover, a separate study validated these findings by showing that, at a concentration of 3 µM, cannabidiol reduced cell invasion and promoted apoptosis in the A549 cell line [284]. In vivo analysis of cannabidiol demonstrated decreased cell proliferation and migration when cannabidiol at a dose of 5 mg/kg was administered to nude mice with xenograft tumors. The researchers attribute these findings to an observed increase in COX-2, peroxisome proliferator-activated receptor-γ (PPAR-γ), and intercellular adhesion molecule-1 (ICAM-1) expression [283].

##### Cypripedin

Cypripedin (Figure 8) is a phenanthrenequinone derived from the orchid *Dendrobium densiflorum* and has been reported to have antiproliferative, anti-inflammatory, antimicrobial, and antioxidant effects [394]. In lung cancer, cypripedin has been shown to inhibit proliferation of H23 cells at a concentration of 50 μM. The researchers reported these effects to be mediated by inhibiting N-cadherin, vimentin, and the Akt/GSK-3β signaling pathway [285]. In lung cancer cell line H460, 50 μM of cypripedin decreased cell proliferation. The investigators suggested these findings to be mediated by inhibition of Bcl-2 expression [286].

##### Daucosterol

Daucosterol (Figure 8) is a steroidal saponin derived from *Grewia tiliaefolia* and has several pharmacological activities, such as antidiabetic, antioxidant, hypolipidemic, anticancer, immunomodulatory, anti-inflammatory, and neuroprotective actions [395]. When applied to A549 cells, daucosterol was found to increase apoptosis and inhibit the cell cycle at the G2/M phase. The researchers believe these anticancer effects to be mediated by downregulation of Bcl-2 expression, with concurrent upregulation of Bax and caspase-3 cleavage [268].

##### Emodin

Emodin (Figure 8) is a natural compound belonging to the anthraquinone family that can be derived from *Polygonum cuspidatum*, *Rheum palmatum*, and *Polygonum multiflorum*; it acts as a tyrosine kinase inhibitor and suppresses tumor growth and cancer cell transformation [396]. Emodin demonstrates anticancer potential against lung neoplasias through its actions on A549 and H1299 cells. Researchers observed that emodin caused endoplasmic reticulum (ER) stress-mediated apoptosis by the tribbles homolog 3 (TRIB3)/NF-κB pathway at concentrations of 20, 40, 60, and 80 μM [287]. Furthermore, te researchers went on to analyze these effects in an in vivo model in nude mice with xenografted tumors, where emodin was again found to initiate ER stress-mediated apoptosis when administrated at a dose of 50 mg/kg [287].

##### Glossogin

Glossogin (Figure 8) is derived from *Glossogyne tenuifolia* and has shown anticancer activity against breast and liver cancer cell lines [397]. In an in vitro analysis of glossogin against A549 lung cancer cells, researchers demonstrated its ability to decrease cell proliferation at a concentration of 12.5 μg/mL. The investigators attributed these findings to an increase in cytochrome c, caspase-3, caspase-9, and the Bak/Bcl-2 ratio [288].

##### Hypericin

Hypericin (Figure 8) is an anthraquinone derivative derived from *Hypericum perforatum* and has been shown to possess anti-inflammatory effects and inhibit various oncogenic signaling molecules, ultimately causing reduced angiogenesis, adhesion, and mitochondrial thioredoxin [168]. In an in vivo study, hypericin enhanced siRNA transfection and reduced HIF-1α, resulting in decreased cell proliferation and angiogenesis in a BALB/c nude mouse tumor model at a dose of 0.1 mg/kg [168,311]. Additional research conducted in a W256 tumor rat and mouse model demonstrated the ability of hypericin to initiate apoptosis at a dose of 2 mg/kg. The researchers did not report any mechanism of action [168,312].

##### Ouabain

Ouabain (Figure 8) is a cardiac glycoside derived from ripe seeds of *Strophanthus gratus* and bark of *Acokanthera ouabaio*, having therapeutic potentials in the management of hypertension, arrhythmia, and heart failure [398,399]. In A549 and H1975 lung cancer cells, ouabain was shown to reduce cell proliferation at a concentration of 25 nM. The researchers believe these results to be mediated by inhibition of JNK and Bcl-2 expression in A549 lung cancer cell lines [289].

##### Physalin A

Physalin A (Figure 8) is an active withanolide derived from *Physalis alkekengi*. It has several biological activities, such as anticancer, antiparasitic, anti-inflammatory, antimicrobial, antiviral, and antinociceptive effects [400]. In lung cancer, physalin A was shown to decrease cell migration, angiogenesis, migration, and proliferation via the JAK/STAT3 signaling pathway in H292, H358, and H1975 cell lines. Following their in vitro analysis, the researchers evaluated the effects of 40 or 80 mg/kg of physalin A in a xenograft mouse model and again found a reduced JAK/STAT3 signaling pathway, with associated reductions in cell proliferation, migration, invasion, and angiogenesis [290].

##### Rhein

Rhein (4, 5-dihydroxyanthraquinone-2-carboxylic acid, Figure 8) is an anthraquinone glycoside abundantly present in several plant species including *Rheum palmatum*, *Polygonum multiflorum* Thunb, *Cassia tora*, and *Aloe barbadensis* Miller. Pharmacological properties of rhein include its ability to cause cell cycle arrest and DNA damage to tumor cells in several cancer models [401]. When applied to A549 cells at a concentration of 45 μM, rhein was shown to inhibit cell proliferation, migration, and invasion. The researchers noted these findings to be associated with an increase in G0/G1 phase cell cycle arrest and p53, p21, and Bax expression [291,292]. In another study, rhein caused apoptosis by reduction of p-PI3K, Akt, mTOR, and Bcl-2 expression at a 100 μM concentration in A549 cells [293]. Moreover, rhein was again demonstrated to inhibit cancer cell proliferation, angiogenesis, and metastasis when applied to PC-9, H460, and A549 cell lines at concentrations of 24.59 µM, 52.88 µM, and 23.9 µM, respectively. The researchers attribute these findings to upregulation of G2/M phase cell cycle arrest and decreased STAT3 and Bcl-2 expression [294]. In a follow-up in vivo study, rhein was again shown to reduce the expression of STAT3 and promote G2/M phase cell cycle arrest at a dose of 60 or 100 mg/kg in a xenograft mouse model [294].

##### Withanone

Withanone (Figure 8) is a steroidal lactone also derived from *Withania somnifera* with an array of therapeutic applications, including anticancer effects [402] and activity against SARS-CoV-2 [403]. In one study utilizing A549 lung cancer cells, withanone was found to decrease cell proliferation, migration, invasion, and metastasis. The researchers noted these findings to be accompanied by reduced expression of CDK2, CDK4, cyclin D and cyclin E, and mortalin and increased p53 and CARF expression [269].

### 7.2. Clinical Studies

While preclinical studies have sparked interest in phytochemicals as agents against lung cancer, clinical trials are required to validate their effects and change the current practice of medical oncology. Most naturally derived agents, such as vincristine, vinblastine, and camptothecin, against lung cancer represented in clinical studies are FDA approved for at least one form of cancer. Two exceptions, however, are resveratrol and seliciclib. Resveratrol is a polyphenol found in berries, grapes, red wine, and peanuts that has been reported to possess antioxidant, antidiabetic, anti-inflammatory, and anticancer effects [404]. In a clinical trial carried out on 96 lung cancer patients, resveratrol suppressed tumor growth by downregulating Forkhead box C2 (FOXC2) and upregulating miR-520h-mediated PP2A/C expression, thereby causing apoptosis in lung cancer cells [405]. Seliciclib is a natural compound present in radish (*Raphnus sativus* L.). Seliciclib (R-roscovitine), a drug in the family of cyclin-dependent kinases, is reported to be effective in treating cancers, inflammation, neurodegenerative diseases, viral infections, glomerulonephritis, and polycystic kidney disease [406]. In a separate, phase II trial, seliciclib (R-roscovitine) was shown to cause decreased tumor growth in lung cancer cells. The researchers attribute this finding to its role in inhibiting RNA-polymerase-II-dependent transcription, leading to the downregulation of myeloid leukemia 1 (Mcl-1) protein [407].

Although use of camptothecin has not been directly approved by the FDA since its isolation from *Camptotheca acuminata*, several of its analogs have received approval for cancer therapy [408]. In a phase I study on cancer patients, camptothecin was documented to synergize with topoisomerase I and DNA, resulting in hindered reassembly of single-strand DNA by intercalating between its nitrogenous bases, leading to the inhibition of bonds at the sites of nicks, thereby harming the structure of the double-stranded DNA chain. Several vinca alkaloids have been approved by the FDA for cancer treatment. Members of this family, including vincristine, vinblastine, vinorelbine, vindesine, and vinflunine, are derived from the Madagascar periwinkle plant [409] and were shown to prevent the polymerization of microtubules, leading to metaphase arrest and cell death in a phase II clinical trial [410]. Paclitaxel and docetaxel were reported to bind with tubulin and disrupt microtubule dynamics, thereby causing mitotic arrest and cell death [411]. In another phase II clinical study, paclitaxel and docetaxel were documented to have response rates of 21–24% and 28–38%, respectively, in advanced non-small lung cancer cell patients, who were first treated with cisplatin [412]. In another phase II clinical study, paclitaxel was reported to promote G2/M phase cell cycle arrest in 37 patients with stage III lung cancer. Paclitaxel was administrated at a concentration of 225 mg/m^2^ for three weeks and although mild hematologic toxicity was observed, adverse events were well tolerated overall [413].

Despite strong support in favor of natural compounds against the development and progression of lung cancer, there is an obvious need for additional clinical trials. Future trials should strive to evaluate the effects of a phytochemical compared to current, first-line treatments. Besides lack of data, other barriers exist before phytochemicals may see widespread use. Taxol, in a phase II clinical study, exhibited cytotoxic symptoms against healthy tissue [414]. Therefore, there is a need to better evaluate the safety profile of phytochemicals, even if their toxicity profiles tend to be better than those of current therapies. Ultimately, the success of these plant-derived anticancer agents as FDA-approved therapies highlights the potential of future drug discovery. The review of the available literature, however, underscores the need for additional studies of underinvestigated compounds.

## 8. Conclusions and Future Perspectives

Lung cancer is the foremost reason for mortality in cancer patients [7]. Despite current chemotherapeutic options, many patients are left without efficacious treatments and suffer as a result. And for those receiving treatment, they must endure numerous side effects. Accordingly, there is an obvious need for the development of safe and effective pharmacons against lung cancer, and the evidence outlined in this review provides strong rationale for further investigation into phytochemicals as potential chemotherapeutic agents. Previously published reviews on phytochemicals targeting lung cancer involved fewer phytochemicals and were not utterly comprehensive or did not incorporate all types of preclinical and clinical studies. Furthermore, a detailed discussion of the preclinical and clinical studies, and molecular mechanisms outlined therein, was previously lacking. Therefore, this comprehensive review serves to explore the anticancer potential of bioactive compounds in lung cancer, along with their molecular mechanisms, as a critical analysis of currently available in vitro, in vivo, and clinical evidence for their role in mitigating lung cancer.

**Figure 9:**
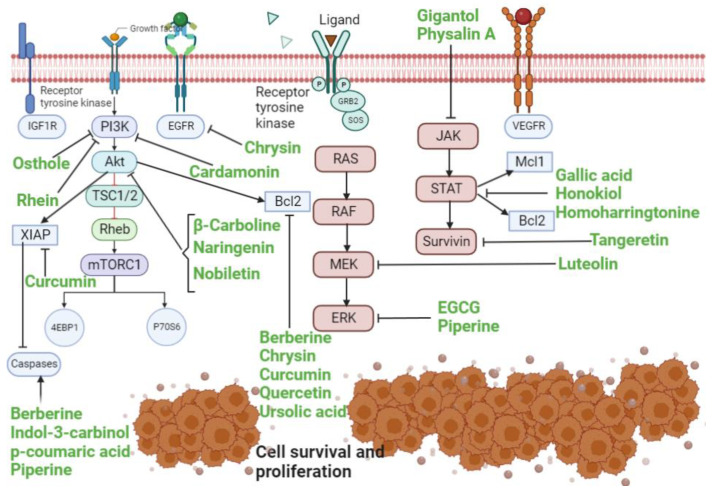
Reported therapeutic targets of select phytochemicals against lung cancer. Binding of ligand to any RTK like EGFR, VEGFR, IGF-1R, HER2, c-MET, or RET results in activation of a multitude of downstream signaling pathways. PI3K/Akt/mTOR pathway incites the activation of effector proteins like 4EBP1 and p70S6K and prosurvival oncogenes including Bcl-2 and XIAP that inhibit the actions of apoptotic caspases. The PI3K/Akt/mTOR pathway also blocks the actions of proapoptotic FOXO. The RAS/RAF/MEK/ERK pathway results in the activation of proto-oncogenes like c-Myc and c-Jun. The JAK/STAT pathway results in the activation of prosurvival oncogenes like Bcl-2, Mcl-1, and survivin. Each of these pathways results in cell survival and proliferation in lung cancer. Signaling molecules and effector proteins of these major pathways of lung cancer serve as targets for phytochemicals. Abbreviations: Bcl-2, B cell lymphoma protein-2; c-MET, c-mesenchymal–epithelial transition factor; 4EBP1, 4E-binding protein 1; EGFR, epidermal growth factor receptor; FOXO, Forkhead box O; HER2, human epidermal growth factor receptor 2; IGF-1R, insulin-like growth factor-1 receptor; Mcl-1, myeloid cell leukemia sequence 1; p70S6K, p70S6 kinase 1; RET, rearranged during transfection; RTK, receptor tyrosine kinase; VEGFR, vascular endothelial growth factor receptor; XIAP, X-linked inhibitor of apoptosis protein (created with BioRender.com, accessed on 28 July 2023).

Phytochemicals are widely reported for their anticancer potential against lung cancer and have been a robust area of research for many years. Several plant metabolites belonging to the class of secondary metabolites such as alkaloids, sulfur-containing compounds, phenolics, and terpenoids are well documented to have antitumor activity or decrease cancer progression. These agents function by modulating various signaling pathways, inducing apoptosis, inhibiting angiogenesis, promoting the disruption of the mitochondrial membrane, and regulating transcription factors and oxidative stress. Several cancer signaling pathways, such as ERK1/2, Akt/mTOR, TGF-β, MAPK, JAK/STAT, NF-κB, and Akt/GSK-3β signaling pathways, have been affected by a range of phytochemicals in various lung cancer models (summarized in Figure 9). Most of the phytochemicals act through complex mechanisms, induce apoptosis and/or inhibit tumor growth, and ultimately play an important role in the cell proliferation and survival. The present review highlights the potential of phytochemicals as promising candidates for cancer prevention and treatment. By targeting multiple cancer hallmarks through various signaling pathways, phytochemicals demonstrate their ability to halt tumor growth and progression. However, further research is needed to fully comprehend the mechanisms and optimize the clinical applications of these natural compounds in cancer therapy.

Notably, the outcomes of the in vitro, in vivo, and clinical studies are promising, but certain limitations are apparent. Numerous preclinical studies had contradictory outcomes, while some studies did not explore the mechanisms of action of the phytochemicals. Furthermore, several phytochemicals were restricted to one type of study (in vitro, in vivo, or clinical). To overcome these limitations, additional preclinical and clinical studies should be conducted to analyze the efficacy of each phytochemical and to explore the potential role of various combinations of phytochemicals in the treatment of lung cancer. Moreover, their potential as adjuvants or complementary agents alongside conventional cancer treatments presents a compelling avenue for future investigation and development of effective anticancer strategies against lung cancer.

Additional research may also address challenges faced by phytochemical-based treatments. The major drawback associated with phytochemicals is their low bioavailability and rapid metabolism in the human body. This may result in reduced effective uptake, causing deficient targeting and undesirable toxicity when consumed in high enough quantities to produce results. To overcome these problems, there is a need to explore a novel delivery system for the effective delivery of plant metabolites. Some phytochemical studies were conducted outside of lung cancer which have observed beneficial results with nanoparticles [415,416]. Moreover, large-scale, well-designed, high-quality, and multicenter randomized clinical studies comparing phytochemicals to first-line treatments are necessary to validate the safety and clinical efficacy of plant metabolites.

In conclusion, numerous phytochemicals exhibit promising outcomes for the prevention and treatment of lung cancer. This review was constructed with hopes that it may open the door for the development of more effective treatments for individuals suffering with lung cancer. It is our anticipation that the information shared in this article will be useful to researchers exploring unique and non-toxic therapeutic avenues for the management of lung cancer. Notably, based on the available literature, several phytochemicals exhibit remarkable and inspiring potential to meet the ever-growing need to prevent and treat lung cancer.

## Figures and Tables

**Table 1:** Currently available drugs for lung cancer treatment.

Drug/Chemical Moiety	Mechanism of Action	Dose, Frequency, and Route	References
Gemcitabine	Inhibits ribonucleotide reductase resulting in inhibition of DNA synthesis	1000 mg/m^2^, weekly, i.v.	[105,106,107]
Docetaxel	Binds to tubulin protein of microtubules, promotes its polymerization and stabilization, resulting in cell arrest in G2/M phase	75 mg/m^2^, daily, i.v.	[108,109]
Carboplatin	Forms adducts with purine bases, resulting in inhibition of DNA replication and subsequent apoptosis of cancer cells owing to damaged DNA	25 mg/m^2^, daily, i.v.	[110,111]
Cisplatin	Forms adducts with purine, resulting in inhibition of DNA replication and subsequent apoptosis of cancer cells owing to damaged DNA	75 mg/m^2^, daily, i.v.	[108,110]
Trametinib	Inhibits MEK 1/2, resulting in obstruction of the RAS/RAF/MEK/ERK oncogenic pathway and cell cycle arrest	2 mg, daily, orally	[112,113]
Dabrafenib	Inhibits of RAF, resulting in obstruction of the RAS/RAF/MEK/ERK oncogenic pathway and cell cycle arrest	150 mg, twice daily, orally	[112,113]
Atezolizumab	Reverses immunosuppression within the tumor by blocking PD-L1 by binding to its receptor	1200 mg, every 3 weeks, orally	[114,115]
Pembrolizumab	Reverses immunosuppression within the tumor by blocking PD-L1 by binding to its receptor	250 mg, every 3 weeks, orally	[116]
Nivolumab	Reverses immunosuppression within the tumor by blocking PD-L1 by binding to its receptor	240 mg, every 2 weeks, orally	[117]
Selumitinib	Inhibits MEK 1/2, resulting in obstruction of the RAS/RAF/MEK/ERK oncogenic pathway and cell cycle arrest	75 mg, twice daily, orally	[118,119]
Navitoclax (ABT-263)	Blocks binding of Bcl-2 and BCL-XL to BIM, halting the antiapoptotic outcome	150 mg, daily, orally	[37,118,120]
Selpercatinib	Inhibits multiple altered RET kinase isoforms, thus inhibiting oncogenic signaling	20 mg, twice daily, orally	[94,121]
Crizotinib	Induces apoptosis in tumor cells and produces G1/S phase arrest by inhibiting ALK, MET, and ROS1 and downregulating JAK and STAT	250 mg, twice daily, orally	[66,122,123]
Alectinib	Induces apoptosis in tumor cells by inhibiting ALK	300 mg, twice daily, orally	[124]
Ceritinib	Inhibits ALK tyrosine kinase	400 mg, daily orally	[125,126]
Ensartinib	Inhibits ALK tyrosine kinase and oncogenic triggers from MET, ROS1, SLK, ABL, LTK, anexelekto (Axl), and EPHA2	225 mg, daily orally	[127,128,129]
Bevacizumab	Inhibits VEGF, resulting in angiogenesis	15 mg/kg, every three weeks, i.v.	[130,131]
Buparlisib	Inhibits PI3K, resulting in downregulation of PI3K/Akt/mTOR signaling and downstream cancer cell proliferation and angiogenesis	100 mg/day, orally	[132,133]
Cabozantinib	Inhibits c-MET, RET, and VEGFR2 tyrosine kinase receptors, thus obstructing the stimulation of downstream signaling molecules involved in tumor proliferation and angiogenesis	60 mg, daily, orally	[134,135]
Capmatinib	Inhibits c-MET, thus obstructing the stimulation of downstream signaling molecules involved in tumor proliferation	400 mg, twice daily, orally	[136]
Erlotinib	Inhibits EGFR tyrosine kinase, resulting in obstruction of cancer cell proliferation by arresting cells in G0/G1 phase of cell cycle	150 mg, daily, orally	[84,137]
Gefitinib	Inhibits EGFR tyrosine kinase blocking oncogenic signals from EGFR-activating mutations	250 mg, daily, orally	[137]
Afatinib	Inhibits EGFR tyrosine kinase blocking oncogenic signals from EGFR-activating mutations	40 mg, daily, orally	[138]
Rociletinib	Inhibits EGFR tyrosine kinase blocking oncogenic signals from EGFR-activating mutations	625 mg, twice daily, orally	[139,140]
Cetuximab	EGFR inhibition and downregulation, antibody-mediated and complement-mediated cytotoxicity in lung cancer cells	400 mg/m^2^ loading dose followed by 250 mg/m^2^ dose weekly, i.v.	[141]
Emibetuzumab	Inhibits ligand-dependent and ligand-independent MET oncogenic signaling	750 mg, every two weeks, orally	[88]
Napabucasin	Inhibits STAT3 and promotes its downregulation, resulting in the inhibition of oncogenic transducer signaling and triggering apoptosis	240 mg, twice daily, orally	[142,143]

Abbreviations: ABL, Abelson murine leukemia viral oncogene; ALK, anaplastic lymphoma kinase; Axl, anexelekto; Bcl-2, B cell lymphoma 2; BCL-XL, B cell lymphoma-extra-large; BIM, Bcl-2-interacting mediator of cell death; c-MET, c-mesenchymal–epithelial transition factor; EGFR, epidermal growth factor receptor; EPHA2, ephrin type-A receptor 2; ERK, extracellular signal-related kinase; JAK, Janus kinase; LTK, leukocyte receptor tyrosine kinase; MEK, mitogen-activated protein kinase; MET, mesenchymal–epithelial transition factor; mTOR, mammalian target of rapamycin; PD-L1, programmed death ligand-1; PI3K, phosphatidylinositol-3-kinase; RAS, rat sarcoma virus gene; RAF, rapidly accelerated fibrosarcoma; RET, rearranged during transfection; ROS1, c-Ros oncogene 1; SLK, Ste20-like kinase; STAT, signal transducer and activator of transcription; STAT3, signal transducer and activator of transcription 3; VEGF, vascular endothelial growth factor; VEGFR2, vascular endothelial growth factor receptor 2.

**Table 2:** In vitro studies of plant-based bioactive compounds in lung cancer.

Phytochemicals	Cell Lines	Conc.	IC_50_	Anticancer Effect	Mechanisms	References
*Alkaloids*						
Acutiaporberine	95-D	0.003 µM	Not reported	Increased cell death	↑Bak/Bcl-2 ratio	[174]
β-Carboline	A549	1.80 μM	Not reported	Showed cytotoxic activity	↑ERK1/2; ↓Akt/mTOR	[175]
Berberine	A549 and H1299	25, 50, 75, and 100 µM	Not reported	Suppressed tumor cell growth and increased cell death	↓Bcl-2; ↑caspase-3; ↑Bax	[176]
Homoharringtonine	A549 and H1975	2–4 μM	3.7 μM(A549) and 0.7 μM (H1975)	Inhibited tumor cell metastasis	↓JAK1/STAT3	[177]
	A549 and H1299	2 µM	Not reported	Inhibited tumor cell growth and metastasis	↓KRAS; ↓ERK; ↓Akt; ↓STAT3; ↓CDK4; ↓CDK6; ↓p21; ↓RB	[178]
Indole-3-carbinol	H1299	400 μM	449.5 μM	Increased cell death and oxidative stress	↑ROS; ↑caspase-3; ↑caspase-7; ↑caspase-9; ↓Bcl-2	[179]
Melosine B	A549	0.064, 0.32, 1.6, 8, and 40 µM	8.1 μM	Exhibited cytotoxicity and increased cell death	Not reported	[180]
Piperine	A549	50, 100, and 200 μg/mL	122 μg/mL	Inhibited tumor cell growth	↑Bax/Bcl-2 ratio; ↑caspase-3; ↑caspase-9	[181]
	A549	20, 40, 80, 160, and 320 µM	198 µM	Inhibited tumor cell migration and invasion	↓ERK 1/2; ↓SMAD 2; ↓TGF-β	[182]
Solamargine	H1650, H1975, PC9, A549, and H1299	2, 4, and 6 µM	Not reported	Reduced tumor cell growth and increased DNA damage	↑ERK1/2; ↓prostaglandin E2; ↓DNMT1; ↓c-Jun	[183]
Vallesiachotamine and iso-vallesiachotamine	H1299	12.5, 25, 50, 100, and 200 μM	4.24 μM (vallesiachotamine) and 3.79 μM (iso-vallesiachotamine)	Suppressed tumor cell growth and caused DNA damage	↑Apoptosis	[184]
*Phenolics*						
Acacetin	A549	1–5 μM	Not reported	Decreased tumor cell growth and viability	↓Activator protein-1; ↓NF-κB; ↓MLK3; ↓MAPK3/6; ↓p38a; ↓MAPK	[185]
Apocynin	A549	50–1000 μM	890 μM	Decreased tumor cell growth and enhanced cell death	↓Cellular microtubule network	[186]
Baicalein	A549 and H1299	2.5, 10, and 40 μM	Not reported	Reduced tumor cell growth, metastasis, and invasion	↓Cellular ezrin S-nitrosylation	[187]
Batatasin III	H460	25–100 μM	Not reported	Inhibited tumor cell migration and invasion	↓EMT; ↓N-cadherin; ↓vimentin; ↓Akt; ↑E-cadherin	[188]
Caffeic acid	A549	50–1000 μM	Not reported	Reduced tumor cell growth	↓Superoxide level	[189]
Cardamonin	A549 and H460	40 μM	Not reported	Decreased tumor cell growth and increased cell death	↑Caspase-3; ↑Bcl-2; ↑Bax; ↑cyclin D1; ↓CDK4; ↓PI3K; ↓Akt; ↓mTOR	[190]
Cardamonin	A549	0.1, 1, 10, and 30 μM	Not reported	Reduced tumor cell growth and enhanced cell death	↓mTOR; ↓DNA synthesis; ↓p70S6K	[191]
Cardamonin analogs	A549 and NCI-H460	0.05–100 µM	0.445 µM (DHC) and 0.166 µM (DHMC)	Inhibited tumor cell growth	↓NF-κB	[192]
Casticin	A549	1, 5, 10 µM	14.3 µM	Suppressed tumor cell growth and enhanced cell death	↓IL-6; ↓COX-2; ↓MAPK; ↓NF-κB; ↓p65; ↓chemokine gene	[193,194]
Chrysin	A549	25, 50, and 75 µg/mL	55.72 μg/mL	Inhibited tumor cell growth and increased cell death	↑Bax; ↓Bcl-2; ↑caspase-3	[195]
Curcumin	A549	10 µM	Not reported	Decreased tumor cell growth and enhanced cell death	↓Prosurvival antiapoptotic factors; ↓EGFR	[196]
	A-549	10–50 µM	Not reported	Caused DNA damage and G2/M phase cell cycle arrest	↑Caspase-3-induced apoptosis; ↑DNA damage; ↑ER stress	[197]
	NCI-H460	30 μM	Not reported	Suppressed tumor cell growth and enhanced cell death	↑Caspase-3; ↑caspase-8; ↓cyclin-dependent kinase 1	[198]
	CL1–5	1–20 μM	Not reported	Inhibited tumor cell growth and metastasis	↑Activator protein-1; ↓E-cadherin;	[199]
	PC-9	50 μM	Not reported	Enhanced DNA damage, cell death and suppressed tumor cell growth	↑DNA damage; ↓Bcl-2; ↓cyclin D1; ↓CDK2; ↓CDK4; ↓CDK6	[200]
	NCI-H292	5–40 μM	15 μM	Increased cell death and inhibited tumor cell growth	↑Bax; ↑caspase-3; ↑caspase-7	[201]
p-Coumaric acid	A549, NCI-H1299, and HCC827	10–100 µg/mL	37.73 μg/mL (A549); 50.6 μg/mL (H1299); 62.0 μg/mL (HCC827)	Increased cell death	↑Bax; ↓Bcl-2; ↑caspase-3; ↑caspase-9	[202]
	H1993	50–100 μM	Not reported	Reduced tumor cell growth and viability	↓Resistance of tyrosine kinase inhibitor	[203]
EGCG	A549 and H1299	20–300 μM	86.4 µM (A549) and 80.6 µM (H1299)	Inhibited tumor cell proliferation and induced apoptosis	↓NF-κB	[204]
	H1299 and CL-13	10–100 µM	174.9 µM(H1299) and 181.5 µM (CL-13)	Reduced tumor cell proliferation	↑ROS; ↓ NF-κB	[205]
	A549	10, 25, 50, and 100 µM	Not reported	Decreased tumor cell growth	↓Nicotine-induced Akt; ↓ERK1/2	[206]
	A549	12.5, 25, and 50 μM	25 μM	Suppressed tumor cell growth, invasion, migration and increased G2/M phase cell cycle arrest	↑Bax/Bcl-2 ratio	[207]
	A549	0.5 μM	Not reported	Decreased tumor cell growth and increased oxidative stress	↑ Nrf2; ↑ROS	[208]
	A549 and NCI-H23	0.05–500 µM	Not reported	Reduced etoposide resistance and tumor cell growth	↑Nrf2; ↑ROS;	[209]
	H1299, H460 and A549	40 µM	Not reported	Decreased tumor cell growth	↑miR-210	[210]
	H1299 and A549	10, 20, and 40 µM	Not reported	Induced apoptosis	↓PI3K/Akt	[211]
EGCG and luteolin	A549 and H460	30 µM (EGCG) and 10 µM (luteolin)	Not reported	Induced apoptosis	↑p53 mitochondrial translocation; ↑DNA damage	[212]
EGCG and theaflavins	NCI-H460	100µM	Not reported	Inhibited tumor cell proliferation and promoted apoptosis	↑p53; ↓Bcl-2	[213]
Ferulic acid	A549	50–1000 μM	Not reported	Enhanced oxidative stress and decreased cell viability	↓Superoxide anion	[189]
Fisetin	A549	5–20 μM	Not reported	Decreased cell viability and increased cell death	↓PI3K/Akt; ↓mTOR	[214]
	NCI-H460	75 µg/mL	Not reported	Inhibited tumor cell growth and viability	↓β-cell lymphoma-2; ↑Bcl-2; ↑caspase-9; ↑caspase-3	[215,216]
	HCC827-ER	10, 20, 40, 60, 80, 100, 120 μM	Not reported	Inhibited tumor cell growth, viability and increased cell death	↓Axl; ↓MAPK; ↓Akt	[217]
Gallic acid	Calu-6 and A549	10–200 μM	10–50 µM (Calu-6); 100–200 µM (A549)	Inhibited tumor cell growth and enhanced oxidative stress	↓GSH; ↑ROS	[218]
	H1975 and H1993	50 μM	Not reported	Increased cell death	↓Src-mediated STAT3; ↓Bcl-2; ↓cyclin D; ↓NF-κB; ↓IL-6	[219]
Genistein	A549	10 μM	Not reported	Inhibited tumor cell growth and enhanced cell death	↑Caspase-3	[220]
	H3255, H1650, and H1781	25 μM	Not reported	Decreased tumor cell growth and increased cell death	↓DNA binding of NF-κB; ↓COX-2; ↓pAkt; ↓EGFR; ↓PGE2	[221]
	SPC-A-1	20–40 μM	Not reported	Reduced tumor cell growth and increased cell death	↓Bcl-2	[222]
	H460	15–30 μM	Not reported	Suppressed tumor cell growth and increased cell death	↓NF-κB	[223]
Gigantol	A549	25, 50, and 100 µM	Not reported	Inhibited tumor cell growth and increased cell death	↓Ki-67; ↓Bcl-2; ↑Bax; ↑Wnt/β-catenin	[224]
	H460	50 μM	Not reported	Increased tumor cell death	↓EMT	[225]
	H460	20–200 µM	Not reported	Reduced tumor cell proliferation, migration, and invasion	↓PI3K/Akt/mTOR; ↓JAK/STAT	[226]
Hesperidin	A549 and NCI-H358	5–50 μM	50 μM	Increased cell death	↑Apoptosis; ↑mitochondrial disruption; ↑caspase-3; ↑NF-κB	[227]
	H1993	5–100 μM	Not reported	Decreased cell viability and enhanced cell death	↓Resistance of tyrosine kinase inhibitor	[203]
Honokiol	A549 and 95-D	5, 10, or 20 μM	Not reported	Increased cell death	↑Bax; ↑caspase-9; ↑PERK; ↑ER stress; ↓Bcl-2	[228,229]
	A549 and LL/2	10–50 μM	21.1 μM	Reduced tumor cell growth and increased cell death	↓VEGF-A	[230]
Mono-demethylated polymethoxyflavones	H1299	1–30 µM	16.5 μM	Increased cell death	↓iNOS; ↓COX-2; ↓Mcl-1; ↑caspase-3; ↑PARP cleavage	[231]
Indolyl-chalcone derivatives	A549	2.5 μM and 5 μM	2.46 μM	Suppressed tumor cell growth	↑Nrf-2/HO-1	[232]
Isorhamnetin	A549	8 μM and 16 μM	Not reported	Increased cell death and mitochondrial dysfunction	↑Caspase-3	[233]
	A549	25 μM	Not reported	Enhanced mitochondrial dysfunction, cell death and decreased tumor cell growth	↑Caspase-3; ↑PARP	[234]
Kaempferol	A549	10–140 μM	72 μM	Inhibited epithelial–mesenchymal transition and increased cell death	↑EMT; ↓E-cadherin; ↓vimentin	[235]
	A549	25 μM	Not reported	Inhibited tumor cell growth and viability	↓E-Cadherin; ↓vimentin ↓Akt1-mediated phosphorylation; ↓TGF-β1	[236]
	H460	30, 50, and 80 μM	50 μM	Enhanced oxidative stress and cell death	↑Caspase-3; ↑AIF	[237]
Kurarinone	H460	2 µg/mL	5.8 µg/mL	Inhibited tumor cell growth	↓NF-κB; ↓tyrosine kinase	[238]
	H1688 and H146	6.25, 12.5, and 25 μM	12.5 µM (H1688) and 30.4 µM (H146)	Enhanced cell death	↓EMT; ↓MMP-2	[239]
Luteolin	A549	20–80 μM	40.2 μM	Increased G2/M phase cell cycle arrest and cell death	↑Bax; ↑procaspase-9; ↑caspase-3; ↓NF-κB; ↑JNK	[240]
	A549	25–100 μM	42.8 µM	Decreased cell viability and increased cell death	↑Bax; ↑caspase-3; ↑caspase-9; ↑MEK/ERK; ↓Bcl-2	[241]
	A549 and H460	10–100 μM	40 μM	Inhibited tumor cell growth and increased cell death	↑miR-34a-5p via targeting MDM4	[242,243]
	NCI-H460	20–160 μM	Not reported	Decreased cell viability and increased cell death	↓Bad; ↓Bcl-2; ↑caspase-3; ↓Sirt1	[244]
Moscatilin	H460	1 μM	Not reported	Reduced tumor cell growth	↓ERK; ↓EMT; ↓Akt; ↓Cav-1	[245]
Naringenin	A549	25, 50, 100, 200, and 300 µM	Not reported	Reduced tumor cell growth	↓MMP-2; ↓MMP-9; ↓Akt	[246]
	A549	10, 100, and 200 µM	Not reported	Decreased tumor cell growth and enhanced cell death	↑Caspase-3; ↓MMP-3; ↓MMP-9; ↑p38	[247]
Nobiletin	A549 (adriamycin resistant)	50 µM	Not reported	Enhanced cell death	↑Caspase-3; ↓Akt; ↓GSK-3β; ↓β-catenin; ↓MRP1	[248]
Osthol	A549	25, 50, 100, 150, and 200 μM	Not reported	Increased G2/M phase cell cycle arrest and cell death	↑Bax; ↓cyclin B1; ↓p-Cdc2 ↓Bcl-2; ↓PI3K/Akt	[249]
	A549	40 and 80 µM	Not reported	Inhibited tumor cell growth, migration, and invasion	↓MMP-2; ↓MMP-9	[250,251]
	A549	5–80 μM	Not reported	Inhibited tumor cell growth and metastasis	↓TGF-β-induced EMT; ↓NF-κB; ↓Snail	[252]
Phloretin	A549, Calu-1 H838, and H520	25–75 μg/mL	Not reported	Enhanced cell death	↓Bcl-2; ↓MMP-2; ↓MMP-9; ↑caspase-3; ↑caspase-9	[253]
	A549	25, 50, 100, and 200 μM	Not reported	Inhibited tumor cell growth and increased cell death	↑Bax; ↓Bcl-2; ↑caspase-3; ↑caspase-9; ↑ERK; ↑JNK; ↑p38; ↑MAPK; ↑JNK1/2; ↓NF-κB	[254]
Polydatin	A549 and NCI-H1975	50 µM	2.95 µM (A549) and 3.23 µM (NCI-H1975)	Reduced tumor cell growth and increased cell cycle arrest	↑Bak/Bcl-2 ratio	[255]
Pterostilbene	NCI-H460 and SK-MES-1	10–100 μM	Not reported	Decreased cell viability and increased cell death	↑Caspase-3; ↑caspase-7	[256]
Quercetin	A549	0.74–4.40 μM	1.41 μM	Decreased cell growth and increased cell death	↑Bax; ↓Bc1-2	[257]
Resveratrol	A549	20 μM	Not reported	Inhibited tumor cell growth and invasion	↓TGF-β1-induced EMT	[258]
	A549	4–64 μM	8.9 μM	Reduced tumor cell growth and increased cell death	↑Caspase-3	[259]
	H1993	1–10 μM	Not reported	Decreased cell viability and increased cell death	↓Resistance of tyrosine kinase inhibitor	[203]
Salicylic acid	A549	1.5–9.5 mM	6.0 mM	Showed cytotoxicity and suppressed tumor cell growth	Not reported	[260]
Tangeretin derivative	CL1-5, H1299, H226, and A549	2.5 and 5 µM	3.2 µM (CL1-5), 6.7 µM (H1299), 10.2 µM (H226), and 9.8 µM (A549)	Enhanced G2/M phase cell cycle arrest, cell death, mitochondrial dysfunction and reduced tumor cell growth	↑Caspase-3; ↓Bcl-2; ↓survivin; ↓PI3K/Akt/mTOR	[261]
Tatariside B, C, and D	A549	0.001, 0.01, 0.1, 1, 10, and 100 µg/mL	18.31 µg/mL (Tatariside B), 6.44–7.49 μg/mL (Tatariside C), and 2.83 μg/mL (Tatariside D)	Enhanced cytotoxicity, oxidative stress, cell death and reduced tumor cell growth	Not reported	[262]
Sulfur-containing compounds						
Allicin	A549 and NCI-H460	10–60 μg/mL	25 µg/mL (A549) and 15 µg/mL (NCI-H460)	Inhibited tumor cell growth	↓Cadherin 2; ↑cadherin 1	[263]
Sulforaphane	H1299, 95-C and 95-D	1–5 μM	9.52 μM (H1299), 9.04 μM (95-C), and 17.35 μM (95-D)	Reduced tumor cell growth and increased S/G2–M phase cell cycle arrest	↓miR-616-5p levels; ↓GSK3β/β-catenin	[264]
	A549 and H1299	0, 5, 10, and 15 mM	Not reported	Inhibited tumor cell growth and enhanced G2/M cell cycle arrest	↑Apoptosis; ↓histone deacetylase	[265]
	A549	2.5 and 5 μM	Not reported	Suppressed tumor cell growth and increased G1/S cell cycle arrest	↓miR-21; ↓CDH1; ↓DNMTs	[266]
*Terpenoids*						
Abietane diterpene	NCI-H460, and A549	10 and 30 µg/mL	14 µM (NCI-H460) and 30 µM (A549)	Enhanced cell death	↑Caspase-3; ↓caspase-9	[267]
β-Sitosterol	A549	50–200 μg/mL	95.19 μg/mL	Enhanced G2/M phase cell cycle arrest	↑Apoptosis	[268]
Cucurbitacin B	A549	10 µM	Not reported	Reduced tumor cell growth and increased cell death	↓CDK2; ↓CDK4; ↓cyclin D; ↓cyclin E; ↓mortalin; ↓hnRNP-K; ↓MMP-2; ↓fibronectin; ↑p53; ↑CARF	[269]
Dihydroartemisinin	PC-14	1 μg/mL	Not reported	Increased cell death	↑p38 MAPK; ↑Ca^2+^	[270]
	LLC cells	5, 10, 20, and 40 µg/mL	26.98 µg/mL	Enhanced G0/G1 phase cell cycle arrest	↑p38 MAPK	[271]
	A549 and H1299	0.23–749.90 µM	80.89 uM	Reduced tumor cell growth	↓Transferrin receptor	[272]
Oridonin	H1975	10 µM	Not reported	Decreased tumor cell metastasis and angiogenesis	↓Mesenchymal transition; ↑proapoptotic activity	[273]
	A549	10, 20, and 30 μM	Not reported	Reduced tumor cell metastasis and angiogenesis	↑Bax; ↑cisplatin-induced apoptosis via AMPK/Akt/mTOR; ↑PARP expression	[274]
Soyasapogenol	H-1299	2–10 µM	6 µM	Reduced tumor cell growth, metastasis and increased cell death	↓CDK2; ↓CDK4; ↓cyclin A; ↓cyclin D1; ↓pATR-Chk1 ↓catenin/vimentin/hnRNPK-mediated EMT	[275]
Thymoquinone	LNM35	1–100 µM	50–78 µM	Suppressed tumor cell growth and increased cell death	↑Caspase-3	[276]
Ursolic acid	A549	11, 22, 44, and 88 µM	Not reported	Decreased cell viability and enhanced autophagy	↑LC3-II/LC3-I ratio; ↑p62; ↑PINK1; ↑Nrf2; ↑ROS; ↓p-Akt/mTOR	[277]
	H1975	0.001–0.1 µM	Not reported	Reduced tumor cell growth and angiogenesis	↓N-cadherin; ↓MMP-2; ↓MMP-9; ↓TGF-β1; ↑E-cadherin	[278]
	A549, H460, H1975, H1299, H520, H82, LLC, and H446	5–40 µM	Not reported	Inhibited tumor cell growth and angiogenesis	↓Bcl-2; ↑cleaved PARP; ↑LC3-II; ↓p-S6K T389; ↓p-Akt	[279]
Withaferin A	A549	10 µM	Not reported	Increased cell death, oxidative stress and decreased cell viability	↑ROS	[280]
Miscellaneous compounds						
Cannabidiol	A549 and H460	3 µM	Not reported	Increased cell death	↑ICAM-1	[281,282]
	A549 and H460	1–10 µM	3.47 µM (A549) and 2.80 µM (H460)	Increased cell death	↑ICAM-1; ↑COX-2; ↑PPAR-γ	[283]
	A549	3 µM	Not reported	Enhanced cell death and reduced tumor cell growth	↑MMP-1	[284]
Cypripedin	H23	50 μM	Not reported	Suppressed tumor cell growth	↓N-cadherin; ↓vimentin; ↓Akt/GSK-3β	[285]
	H460	50 μM	Not reported	Inhibited tumor cell growth	↓Bcl-2	[286]
Daucosterol	A549	50–200 μg/mL	17.46 μg/mL	Reduced tumor cell growth and enhanced G2/M phase cell cycle arrest	↓Bcl-2; ↑Bax; ↑caspase-3	[268]
Emodin	A549 and H1299	20, 40, 60, and 80 μM	Not reported	Enhanced cell death	↑ER stress; ↑TRIB3/NF-κB	[287]
Glossogin	A549	12.5 μg/mL	Not reported	Suppressed tumor cell growth	↑Cyt c; ↑caspase-9; ↑caspase-3; ↑Bak/Bcl-2 ratio	[288]
Ouabain	A549 and H1975	25 nM	Not reported	Inhibited tumor cell growth	↑JNK; ↓Bcl-2	[289]
Physalin A	H292, H358, and H1975	5, 10, and 15 μM	Not reported	Decreased tumor cell growth and enhanced cell death	↓JAK/STAT3	[290]
Rhein	A549	25, 50, and 100 μM	45 μM	Enhanced G0/G1 phase cell cycle arrest and cell death	↑ER stress; ↑p53; ↑p21; ↑Bax; ↓Bcl-2; ↓GADD153; ↓cyt c	[291,292]
	A549	25, 50, and 100 μM	100 μM	Inhibited tumor cell growth	↓Bcl-2; ↓p-PI3K; ↓Akt; ↓mTOR	[293]
	PC-9, H460, and A549	30, 60, and 100 µM	24.59 µM (PC-9), 52.88 µM(H460), and 23.9 µM (A549)	Increased G2/M phase cell cycle arrest and cell death	↓Bcl-2; ↑Bax; ↓STAT3	[294]
Withanone	A549	2.5–10 µM	Not reported	Reduced tumor cell growth and increased cell death	↓CDK2; ↓CDK4; ↓cyclin D; ↓cyclin E ↓mortalin; ↓hnRNP-K; ↓MMP-2; ↓fibronectin; ↑p53; ↑CARF	[269]

Symbols and abbreviations: ↑, increased or upregulated; ↓, decreased or downregulated; AIF, apoptosis-inducing factor; Bax, Bcl-2-associated X protein; Bcl-2, B cell lymphoma-2; CARF, calcium-response factor; CDK, cyclin-dependent kinase; COX, cyclooxygenase; DNMT, DNA methyltransferase; EGFR, epidermal growth factor receptor; EMT, epithelial to mesenchymal transition; ERK, extracellular signal-related kinase; GSH, glutathione; GSK3β, glycogen synthase kinase 3β; HO-1, heme oxygenase-1; ICAM-1, intercellular adhesion molecule-1; IL, interleukin; iNOS, inducible nitric oxide synthase; JAK, Janus kinase; MAPK, mitogen-activated protein kinase; Mcl-1, myeloid cell leukemia-1; MEK, mitogen-activated protein kinase; MLK3, mixed lineage kinase 3; MMP-3, matrix metalloproteinase-3; MRP1, multidrug resistance protein 1; mTOR, mammalian target of rapamycin; NF-κB, nuclear factor-κB; PARP, poly (ADP-ribose) polymerase; PERK, protein kinase RNA-like endoplasmic reticulum kinase; PGE2, prostaglandin E2; PI3K, phosphatidylinositol-3-kinase; PPAR-γ, peroxisome proliferator-activated receptor-γ; ROS, reactive oxygen species; STAT, signal transducer and activator of transcription; TGF-β, transforming growth factor-β; VEGF-A, vascular endothelial growth factor-A.

**Table 3:** In vivo studies of plant-based bioactive compounds in lung cancer.

Phytochemicals	Anticancer Model	Dose (Route)	Anticancer Effects	Mechanisms	References
*Alkaloids*					
Berberine	Xenograft athymic nude mouse model	50, 100, and 200 mg/kg (p.o.)	Increased cell death and decreased tumor weight	↓Bcl-2; ↑Bax; ↑caspase-3	[176]
Evodiamine	Xenograft nude mouse model and Lewis lung carcinoma model	10, 20, and 30 mg/kg (p.o.)	Reduction in tumor volume	↑CD8+ T cells; ↓MUC1-C/PD-L1	[302]
Hirsutine	Lung metastasis model in BALB/c mice	25 µM (i.p.)	Decreased tumor weight	↓NF-κB	[303]
Homoharringtonine	Xenograft tumor mouse model	10 mg/kg (p.o.)	Suppressed tumor growth	↓IL-6; ↓JAK1/STAT3	[177]
	Xenograft tumor mouse mode and transgenic carrying the KRAS mutation model	1.25 and 2.5 mg/kg (i.p.)	Inhibited tumor growth	↓Bcl-2; ↑caspase-3; ↑caspase-9	[178]
Solamargine	Xenograft mouse model	4 and 8 mg/kg (p.o.)	Decreased tumor growth	↑ERK1/2; ↓prostaglandin E2; ↓DNMT1; ↓c-Jun	[183]
*Phenolics*					
Apocynin	Xenograft BALB/c mouse model	50 and 100 mg/kg (i.p.)	Suppressed tumor growth	↓Microtubule network	[186]
Baicalein	Xenograft BALB/c nude mice	2.5, 10, and 40 mg/kg (i.g.)	Reduction in tumor volume	↓Cellular ezrin S-nitrosylation	[187]
Cardamonin	Xenograft nude mouse model	5 mg/kg (i.p.)	Enhanced cell death and inhibited tumor cell metastasis	↑Bax; ↓Bcl-2; ↑caspase-3; ↓cyclin D1; ↓CDK4; ↓PI3K; ↓Akt/mTOR	[304]
Chrysin	Tumor reduction model in BALB/c mice	1.3 mg/kg (p.o.)	Increased cell death	Caspase-3	[195]
Curcumin + neoadjuvant radiotherapy	Lung carcinoma model in C57BL/6J mice	100 µg (i.v.)	Inhibited angiogenesis and increased cell death	↓Prosurvival antiapoptotic factors	[196]
p-Coumaric acid	Xenograft model in nude mice	50 mg/kg (i.p.)	Enhanced cell death	↑Bax; ↓Bcl-2; ↑caspase-3; ↑caspase-9	[202]
EGCG	Xenograft BALB/c athymic nude mouse model	20 mg/kg (i.p.)	Inhibited tumor size and induced apoptosis	↓NF-κB	[305]
	Xenograft BALB/c athymic nude mouse model	100 µM (s.c.)	Inhibited tumor number	↓Nicotine-induced Akt; ↓ERK1/2; ↓ HIF-1α; ↓VEGF	[206]
	Xenograft nude mouse model	1.62 mg/kg (i.p.)	Inhibited tumor number and size	↓Cisplatin-induced lung tumorigenesis	[306]
EGCG and luteolin	Xenograft nude mouse model	125 mg/kg (EGCG) and 10 mg/kg (luteolin) (p.o.)	Decreased tumor size, volume and induced tumor cell apoptosis	↑p53 mitochondrial translocation; ↑DNA damage	[212]
Gallic acid	Xenograft tumor mouse model	200 mg/kg (i.p.)	Increased cell death and G2/M phase cell cycle arrest	↓Src-mediated STAT3; ↓Bcl-2; ↓cyclin D	[219]
Gigantol	Xenograft tumor mouse model	Pretreated 20 µM (i.p.)	Inhibited tumor cell growth, migration, and invasion	↓PI3K/Akt/mTOR; ↓JAK/STAT	[226]
Honokiol	Orthotopic model of lung cancer in NOD/SCID mice	7.5, 37.5, and 75 μmol/kg (p.o.)	Decrease in tumor volume	↑ ROS; ↑mitochondrial Prx3 oxidation; ↑AMPK; ↓STAT3	[307]
Kurarinone	Xenograft in BALB/c nude mouse model	100 mg/kg (i.p.)	Increased cell death	↓Bcl-2; ↑caspase-8; ↑caspase-3	[238]
Nobiletin	Xenograft in athymic BALB/c nude mouse model	40 mg/kg (s.c.)	Inhibited tumor growth and enhanced cell death	↑Caspase-3; ↓Akt; ↓GSK3β, β-catenin; ↓MRP1	[248]
Quercetin	Xenograft BALB/c nude mouse model	8 mg/kg (i.v.)	Decreased tumor growth, viability and promoted cell death	↑Bax; ↓Bc1-2	[257]
Resveratrol	Xenograft BALB/c nude mouse model	15, 30, and 60 mg/kg (i.v.)	Inhibited tumor growth and increased cell death	↑Caspase-3	[259]
Tangeretin derivatives	Xenograft BALB/c athymic nude mouse model	20 mg/kg (i.p.)	Increased G2/M cell cycle arrest, mitochondrial disruption, cell death and decreased tumor growth	↓Bcl-2; ↑caspase-3; ↓phophoatidylinositol 3-kinase/Akt/mTOR	[261]
*Sulfur-containing compounds*					
Sulforaphane	Xenograft nude mouse model	9 µM (p.o.)	Suppressed tumor growth and enhanced G2/M cell cycle arrest	↑Apoptosis; ↓histone deacetylase	[265]
Sulforaphane	Xenograft nude mouse model	25 and 50 mg/kg (i.p.)	Reduction in tumor volume	↑E-cadherin; ↑ZO-1; ↑ERK5; ↓N-cadherin; ↓Snail 1	[308]
*Terpenoids*					
Thymoquinone	Xenograft nude mouse model	10 mg/kg (i.p.)	Inhibited tumor growth	↑Caspase-3	[276]
Betulinic acid	Xenograft nude mouse model	50 and 75 mg/kg (i.p.)	Suppressed tumor growth	↓Skp2; ↑p27; ↑E-cadherin	[309]
Scabertopin	Xenograft nude mouse model	20 mg/kg (i.p.)	Inhibited tumor growth	↑Apoptosis ↑Bax; ↑ROS	[310]
Soyasapogenol	Xenograft immune-deficient mouse model	15 mg/kg (i.v.)	Suppressed tumor growth and metastasis	↓CDK2; ↓CDK4; ↓cyclin A; ↓cyclin D1; ↓catenin/vimentin/hnRNPK	[275]
*Miscellaneous compounds*					
Cannabidiol	Xenograft nude mouse model	5 mg/kg (i.p.)	Increased cell death and inhibited tumor proliferation	↑ICAM-1; ↑COX-2; ↑PPAR-γ	[283]
Emodin	Xenograft model in nude mice	20 and 50 mg/kg (i.p.)	Induced cell death	↑ER stress; ↑TRIB3/NF-κB	[287]
Hypericin	Rodent tumor model in BALB/c nude mice	0.1 mg/kg (i.p.)	Displayed antiproliferative effects	↑siRNA; ↓HIF-1α	[168,311]
	Rodent tumor model/W256 tumor rats and mice	2 mg/kg (intra tumor)	Inhibited tumor proliferation and induced cell death	↑Apoptosis	[168,312]
Physalin A	Xenograft mouse model	40 and 80 mg/kg (i.p.)	Decreased tumor growth and increased cell death	↓STAT3; ↓JAK/STAT3	[290]
Rhein	Xenograft mouse model	60 and 100 mg/kg (i.p.)	Increased G2/M phase cell cycle arrest, cell death and reduction in tumor volume	↓Bcl-2; ↑Bax; ↓STAT3	[294]

Symbols and abbreviations: ↑, increased or upregulated; ↓, decreased or downregulated; Bax, Bcl-2-associated X protein; Bcl-2, B cell lymphoma-2; CDK, cyclin-dependent kinase; DNMT, DNA methyltransferase; ERK, extracellular signal-related kinase; GSK3β, glycogen synthase kinase 3β; JAK, Janus kinase; MRP1, multidrug resistance protein 1; mTOR, mammalian target of rapamycin; MUC1, mucin 1; NF-κB, nuclear factor-κB; PI3K, phosphatidylinositol-3-kinase; ROS, reactive oxygen species; STAT, signal transducer and activator of transcription; ZO-1, zonula occludens-1 epithelial marker.
